# Seroepidemiology of human leptospirosis in subtropical Bhutan, 2024

**DOI:** 10.1371/journal.pntd.0014821

**Published:** 2026-11-04

**Authors:** Monu Tamang, Rie Takeuchi, Thinley Dorji, Kinley Choki, Tshokey Tshokey, Chenga Dawa, Choki Dolkar, Kesang Wangchuk, Dorji Tshering, Sangay Tenzin, Kinley Wangchuk, Rinzin Wangchuk, Naoko Yamamoto, Yasuyuki Kato

**Affiliations:** 1 Department of Global Health and Infectious Diseases, Graduate School of Public Health, International University of Health and Welfare, Narita, Japan; 2 Health and Medical Research Centre, Central Regional Referral Hospital, Gelephu, Bhutan; 3 Department of Internal Medicine, Central Regional Referral Hospital, Gelephu, Bhutan; 4 Department of Pharmacy, Central Regional Referral Hospital, Gelephu, Bhutan; 5 Faculty of Postgraduate Medicine, Khesar Gyalpo University of Medical Sciences of Bhutan, Thimphu, Bhutan; 6 Department of Pathology and Laboratory Medicine, Jigme Dorji Wangchuck National Referral Hospital, Thimphu, Bhutan; 7 Department of Laboratory Medicine, Central Regional Referral Hospital, Gelephu, Bhutan; 8 Ministry of Health, Royal Government of Bhutan, Thimphu, Bhutan; 9 Royal Centers for Disease Control, Thimphu, Bhutan; 10 Department of Clinical Nursing, Central Regional Referral Hospital, Gelephu, Bhutan; 11 Department of Infectious Diseases, School of Medicine, International University of Health and Welfare, Narita, Japan; Federal Institute for Risk Assessment: Bundesinstitut fur Risikobewertung, GERMANY

## Abstract

**Background:**

Leptospirosis causes approximately 1.03 million cases and 58,900 deaths annually worldwide yet remains underrecognized because infections are often mild or self-limiting and require serological confirmation. In Bhutan, limited diagnostic capacity constrains surveillance, and data on human exposure in high-risk subtropical areas are limited. This study aimed to estimate seroprevalence, identify circulating serovars, map geographic distribution of seropositive cases, and assess potential risk factors for *Leptospira* exposure among adults in subtropical Bhutan.

**Methods/principal findings:**

We conducted a cross-sectional study during September–October 2024 in six subtropical communities in southern Bhutan. A total of 503 adults were enrolled using probability-proportional-to-size (PPS) sampling; 492 were included in the final analysis after excluding 11 participants for inadequate blood sample or hemolysis. Serum samples were tested by IgM and IgG ELISA and by microscopic agglutination test (MAT), the reference standard used to define seropositivity for potential risk factor analysis and primary reacting serovar identification. MAT-based seroprevalence was 19.3% (95% CI: 16.0 – 23.1), compared with 22.4% (95% CI: 18.8 – 26.4) by ELISA IgM and 16.1% (95% CI: 13.0 – 19.7) by ELISA IgG. Among MAT-positive samples (n = 95), the predominant serovars were Australis (49%) and Lai-like (13%). Gelephu Thromde had the highest serovar diversity, while Australis and Patoc were detected in all six study areas. Multivariable logistic regression identified paddy farming (adjusted OR: 4.74; p < 0.001) and drinking unboiled water (adjusted OR: 2.34; p = 0.002) as independently associated with MAT seropositivity. In addition, residence in crowded urban (adjusted OR: 4.08; p = 0.001) or urban areas (adjusted OR: 2.94; p = 0.002) relative to rural areas and lower education (primary education: adjusted OR: 2.52; p = 0.013; unable to read and write: adjusted OR: 2.02; p = 0.034) were associated with MAT seropositivity.

**Conclusion/significance:**

Approximately one in five adults in subtropical Bhutan had serological evidence of prior *Leptospira* spp. exposure, indicating that human exposure is more common than previously recognized. Given the cross-sectional design, these associations cannot establish temporality and self-reported exposure to potential risk factors may be subject to recall bias. These findings, however, provide baseline data on population exposure and point to actionable public health interventions: (1) educate communities on preventive behaviors, (2) promote use of protective gear during high-risk activities, (3) expand research and surveillance capacity to better understand the epidemiology of leptospirosis and (4) strengthen hospital-based diagnostics to enable early treatment and reduce complications from leptospirosis in Bhutan.

## Author summary

Leptospirosis is a zoonotic disease caused by *Leptospira* spp. The bacteria are harbored by a wide range of mammals, particularly rodents. It remains an under-recognized public health challenge in the subtropical region of Bhutan. This study investigated seroprevalence and associated potential risk factors in six communities in subtropical Bhutan. We found that 19.3% of participants were seropositive for *Leptospira* antibodies, indicating that approximately one in five people had evidence of prior exposure. Among the potential risk factors, people engaged in paddy farming and those who reported drinking unboiled water in 12 months preceding the study had higher odds of being seropositive. These findings should inform public health interventions, including educating communities on preventive behaviors, promoting use of protective gear during high-risk activities and expanding research and surveillance capacity for leptospirosis in Bhutan.

## Introduction

Leptospirosis is a zoonotic disease caused by spirochetes of the genus *Leptospira*. Harbored and excreted by diverse mammalian hosts, the bacteria spread directly through urine or aborted tissues and indirectly through contaminated water or soil [1], resulting in an estimated 1.03 million cases and 58,900 deaths annually [2]. While the infection is predominantly asymptomatic or presents with mild, non-specific, and self-limiting symptoms, about 10% of cases progress to severe Weil’s syndrome with organ failure [3], with case-fatality ratios ranging from 5 to 15% in severe disease and exceeding 50% in rare pulmonary hemorrhagic syndrome [2]. Leptospirosis is a biphasic disease characterized by an initial, acute (leptospiremic) phase, during which *Leptospira* circulate in the bloodstream, followed by an immune (leptospiruric) phase marked by antibody production and potential organ involvement. The acute phase of leptospirosis responds to low-cost antibiotics, including penicillin, cephalosporins, doxycycline and macrolides and early treatment is critical to prevent severe, often fatal complications [3,4].

In Bhutan, the first documented evidence of human leptospirosis was a suspected outbreak in the southwest district of Chukha in 2008 [5]. Also, from 2008 to 2018, the disease was designated notifiable, with sporadic cases continually detected through sentinel surveillance of acute undifferentiated febrile illnesses. In addition, in June and July 2025, Bhutan experienced its largest recorded leptospirosis outbreak, resulting in 143 hospitalizations and three deaths [6]. Although enzyme-linked immunosorbent assay (ELISA) and microscopic agglutination test (MAT) are standard diagnostic methods [7], serodiagnostic facilities in Bhutan are limited to the Royal Centers for Disease Control (RCDC) and the Jigme Dorji Wangchuck (JDW) National Referral Hospital in Thimphu, with the latter facing an erratic supply of test kits. As of December 2025, polymerase chain reaction (PCR) for leptospirosis was not available in the country. The absence of reliable point-of-care (POC) diagnostics [8,9], combined with clinical testing that favors known or highly endemic febrile pathogens such as dengue [10], leads to underdiagnosis and under-reporting, particularly in areas with low awareness and low endemicity [11]. As climate change increasingly influences outbreak patterns [12], monitoring longitudinal changes in seroprevalence is essential to anticipate disease dynamics and develop evidence-based public health strategies.

Although leptospirosis has been reported in both humans and animals in Bhutan [13,14], critical epidemiological uncertainties remain. The extent of population exposure, the diversity of circulating serovars, and the factors associated with transmission have not been characterized, particularly in subtropical settings where environmental conditions favor *Leptospira* persistence. No previous human seroprevalence study has been conducted in the study area, which is also endemic for other tropical diseases, including dengue, malaria, and scrub typhus, for which leptospirosis is an important differential diagnosis. In addition, although dogs are vaccinated against leptospirosis, vaccination for livestock and other companion animals is not currently practiced in Bhutan. These evidence gaps hinder risk assessment, surveillance and design of targeted prevention and control measures.

Given the favorable environmental conditions for *Leptospira* transmission in southern Bhutan and the limited epidemiological data available, we hypothesized that adults living in subtropical communities would show measurable serological evidence of exposure to *Leptospira* spp. and that environmental and occupational contact with potentially contaminated water or soil would be associated with seropositivity. Specific aims of the study were to (1) estimate human leptospirosis seroprevalence, (2) identify circulating serovars, (3) map the seropositive cases, and (4) assess potential risk factors for seropositivity.

## Methods

### Ethics statement

The study was approved by the Research Ethics Board of Health, Bhutan (PO/RL/2024.13.MC) and the Institutional Review Board of the International University of Health and Welfare, Japan (23-Ac-1). The Royal Centers for Disease Control in Bhutan granted the research site clearance, and administrative clearance was obtained from the Ministry of Health, Sarpang District Administration and Gelephu Thromde. All participants provided written informed consent prior to participation. For those unable to read or write, consent was given via thumb impression, with a witness signing on their behalf.

### Study design and setting

We conducted a cross-sectional study to examine the seroprevalence of leptospirosis in Sarpang district, a subtropical region in south-central Bhutan bordering the Indian state of Assam (Fig 1). The study area covered five sub-districts and one municipality – Chhudzom, Jigme Chhoeling, Senggye, Shompangkha, Gelephu Thromde and Umling. Geographically, the district’s elevation ranges between 200 and 3,600 meters above sea level, and the district experiences heavy monsoon rainfall from May to September, which frequently results in pluvial and fluvial flooding.

**Fig 1:**
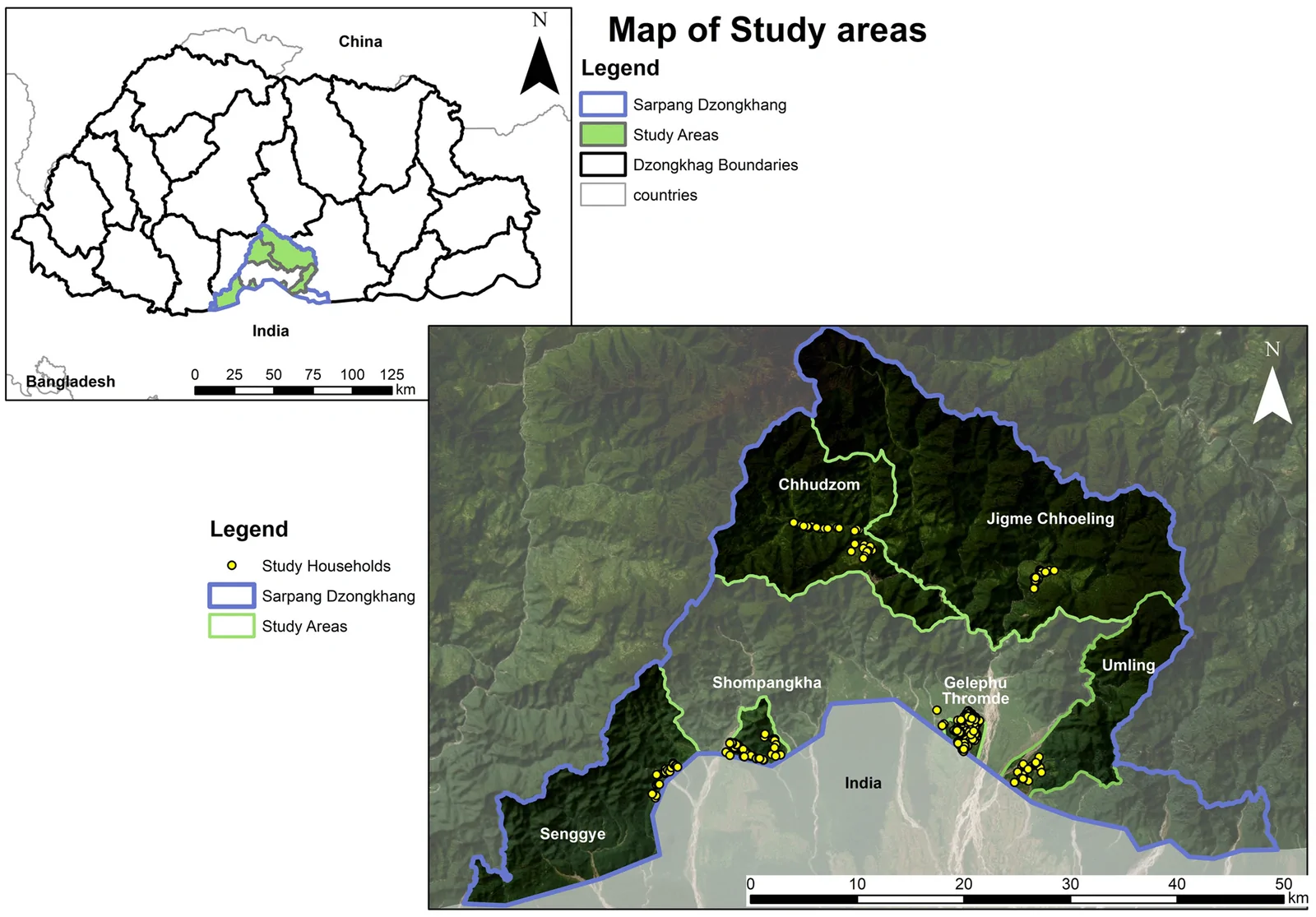
Map of five sub-districts and one municipality with selected households in Sarpang district of Bhutan, 2024. The basemap used for geographic visualization is the Esri ArcGIS 10.8 basemap (World Imagery) used under written permission from Esri (Environmental Systems Research Institute, Inc.) granted on 29 July 2026. Content is the intellectual property of Esri and is used herein with permission. Copyright 2026 Esri and its licensors. All rights reserved. Boundary shapefiles for Bhutan were obtained from the Bhutan National Spatial Data Infrastructure (NSDI) System (https://nsdi.systems.gov.bt/), which are publicly available and in the public domain. The geolocation of the study population and seropositive participants was generated and plotted by the authors using geographic information system (GIS) tools in ArcGIS 10.8.

### Study population

We obtained the current (2024 – 2025) district household registry from District Public Health Office, Sarpang. However, as the household list for Gelephu Thromde (municipality) was unavailable, households in the municipality were estimated from the 2017 Population and Housing Census [15]. The study area comprised **7,705 households distributed across five sub-districts and one municipality.** The sample size was calculated using the single-population proportion formula:


$$\begin{array}{c} n=\frac{Z^{\text{2}}P(\text{1}-P)}{d^{\text{2}}} \end{array}$$

where:

n = required sample size

Z = standard normal deviate (1.96 for 95% confidence)

P = expected prevalence

d = desired precision (margin of error)

Assuming an expected ELISA-based seroprevalence of 17.6% based on a previous study in Bhutan [13], a precision of 5% and a 95% confidence interval, the minimum sample size was estimated at 223 households (one participant per household). A study design effect of 2.0 was applied to account for the cluster-based sampling design, resulting in a required sample size of 446 households. The sample size was further inflated by 5% to account for anticipated non-response and potential sample exclusions, yielding a target sample size of 469 households. A total of 469 households were subsequently selected across the study sites using probability-proportional-to-size (PPS) sampling based on the total households of each site.

### Sample and data collection

To account for anticipated exclusions, a total of 503 samples were collected, exceeding the target sample size of 469. Ultimately, 11 samples were excluded due to hemolysis or insufficient volume, leaving 492 samples that were tested and included in the analysis. Eligible participants had to be ≥ 18 years old and residents of the study areas for a minimum of one year. Exclusion criteria included non-Bhutanese nationals and individuals with severe mental illnesses to ensure the reliability of survey response.

Trained data collectors visited the communities between September 8 and October 27, 2024. We enlisted the local government bodies to disseminate the information about the study in the communities through social media platforms to foster community engagement. Households were selected from the administrative registry using a random sequence generator; in buildings with multiple households, we randomly selected one household, followed by the selection of a single eligible member per household through a lottery draw. If a selected individual declined to provide informed consent, another eligible household member was approached instead; for single-member households, the nearest neighboring household not on the original list served as a substitute. This occurred for 5 households (1.0%), of which 3 were replaced with the next eligible household member and 2 with a neighboring household.

Participants were interviewed using a structured, standardized, pre-tested and closed questionnaire administered in English and Dzongkha (the national language of Bhutan), with explanation provided in local languages when necessary. Data were recorded using Epicollect5 (https://five.epicollect.net/) and included sociodemographic characteristics, geolocation, current symptoms and exposure to potential risk factors for MAT seropositivity. Data completeness was reviewed after each field day, and participants were contacted by phone within 24 hours to obtain any missing information. Settlements were classified as urban and rural according to the National Statistics Bureau of Bhutan [15]. Within urban areas, “crowded urban” was defined as densely settled urban neighborhoods characterized by small, substandard housing and inhabited predominantly by households of low socioeconomic status. Animal exposure was defined as direct contact with rat, rabbit, bat, cattle, goat, pig, dog and cat in the preceding 12 months; environmental exposure was defined as contact with potentially contaminated water sources or agricultural soil during occupational or recreational activities across three domains in the past 12 months: environmental (n = 6), behavioral/occupational (n = 9) and recreational (n = 3) (S2 Table).

After the questionnaire, 5 mL of venous blood was collected from each participant in plain tubes without anticoagulant. When data collectors and participants were of opposite sex, a family member acted as chaperone. Blood samples were stored in the cold chain box during field work. Following the collection, samples were placed in a water bath at 37 °C for 15 minutes for proper clot retraction and centrifuged at 1,800 × g for three minutes. The serum samples were stored at -30 °C at the Central Regional Referral Hospital in Gelephu, before transferring them to RCDC, the national reference laboratory for infectious diseases surveillance, where they were stored at -40 °C until batch testing.

### Serological testing

Serum samples were tested for anti-*Leptospira* IgG and IgM antibodies using commercially available ELISA test kits; *Leptospira* IgG ELISA (Gold Standard Diagnostics, Germany. https://www.goldstandarddiagnostics.com/) and Lepto IgM MICROLISA (J. Mitra & Co. Pvt. Ltd, India. https://jmitra.co.in/product-details/leptospira-igm-microlisa/). The assay procedure strictly adhered to the manufacturers’ instructions. Frozen serum samples were thawed, and test kits were equilibrated at room temperature before testing. Serum samples were diluted using the provided diluent and added to antigen-coated microtiter wells and incubated at 37°C for 30 min for IgM and 60 min for IgG. Following incubation, the wells were washed with wash buffer to remove unbound antibodies. Enzyme-conjugated anti-human IgM and IgG were added, followed by a second incubation at 37°C for 30 min for IgM and at room temperature for 30 min for IgG. After washing, substrate solution was added and incubated in the dark at room temperature for 25–30 min. The reaction was stopped by adding stop solution and optical density (OD) was measured at 450 nm with a reference wavelength of 620–630 nm using a BioTek ELx808 microplate reader. Qualitative detection of *Leptospira* IgM/IgG antibodies was interpreted based on a cutoff value of 10: results > 11 were considered positive, 9 – 11 indeterminate and < 9 negative. According to the manufacturers, the *Leptospira* IgG kit has a sensitivity of 100% and specificity of 97.37%, while the IgM MICROLISA has sensitivity of 99.62% and specificity of 99.92%. Positive and negative controls were included in each run.

Irrespective of ELISA results, all samples were tested using MAT (Fig 2). MAT was performed using Difco *Leptospira* Medium Base EMJH (Becton Dickinson Company, Franklin Lakes, NJ, USA) supplemented according to the manufacturer’s instructions and reagents obtained from Sigma-Aldrich (St. Louis, MO, USA www.sigmaaldrich.com). The MAT, the gold standard serodiagnostic test for leptospirosis, detects both IgM- and IgG-class antibodies [7]. A battery of reference live antigen of 12 pathogenic *Leptospira* serovars and one saprophytic serovar (Table 1) was maintained by serial subculture in supplemented EMJH base medium. The age of culture at the time of testing was 7 – 10 days. Serum samples were serially diluted in phosphate-buffered saline (PBS) from 1:10 dilution to 1:160 and mixed with an equal volume of live antigen suspension. Plates were incubated at 30 °C for three hours and examined for agglutination by dark-field microscopy. Any serovar showing a titer of ≥1: 80 was considered seropositive. For this study, only the primary reacting serovar, defined as the serovar with the highest titer, was recorded. Quality control procedures included the use of positive and negative control sera and routine verification of culture viability before testing. Given the absence of population-specific MAT reference titers for Bhutan and the study’s focus on seroprevalence rather than acute infection, a ≥ 1:80 cutoff was adopted to maximize sensitivity for detecting previous exposure [16].

**Table 1:** Panel of live antigens of *Leptospira* spp. in the microscopic agglutination test at the Royal Centers for Disease Control, Thimphu, Bhutan, 2024.

Serogroup	Serovar	Strain	Genomospecies
Australis	Australis	Ballico	*L. interrogans*
Autumnalis	Bangkinang	Bangkinang I	*L. interrogans*
Canicola	Canicola	Hond Utrecht IV	*L. interrogans*
Grippotyphosa	Grippotyphosa	Moskva V	*L. kirschneri*
Hebdomadis	Hebdomadis	Hebdomadis	*L. interrogans*
Icterohemorrhagiae	Icterohemorrhagiae	RGA	*L. interrogans*
Louisiana	Louisiana	LSU 1945	*L. noguchii*
Pyrogenes	Robinsoni	Robinson	*L. interrogans*
Bataviae	Bataviae	Swart	*L. interrogans*
Pomona	Pomona	Pomona	*L. interrogans*
Sejroe	Hardjo	Hardjoprajitno	*L. interrogans*
Semaranga	Patoc	Patoc I	*L. biflexa*
Icterohemorrhagiae	Lai-like	AF-61	*L. interrogans*

**Fig 2:**
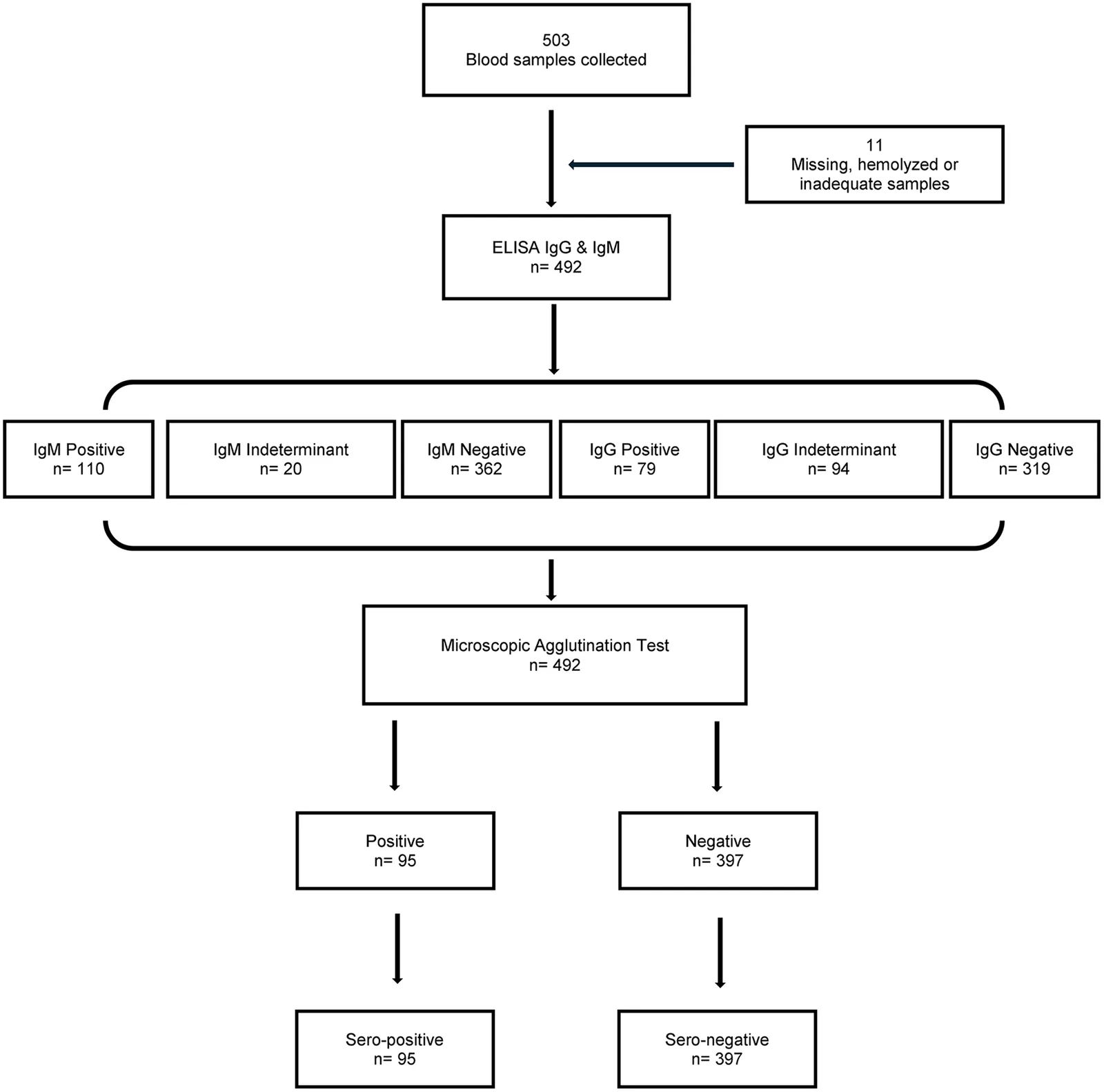
Laboratory testing pathway to define *Leptospira* spp. seropositive and seronegative persons to estimate seroprevalence of human leptospirosis in subtropical Bhutan, 2024.

### Statistical analysis

Sociodemographic and risk factor data were exported from Epicollect5 as a CSV file, while laboratory results were recorded in Excel and integrated for analysis (S1 Data). Descriptive statistics were used to describe sociodemographic characteristics and potential risk factors for seropositivity. Mean and standard deviation (SD) were calculated to summarize continuous variables and frequency and percentage to summarize categorical variables. Associations between potential risk factors and MAT seropositivity were assessed using logistic regression to calculate crude odds ratio (OR) with 95% confidence intervals (CIs). Although both ELISA and MAT were performed, MAT serostatus (seropositive for at least one serovar among 13 serovars tested) was selected for modeling because MAT is considered the gold standard reference test and provides greater specificity for identifying serological evidence of exposure to *Leptospira* spp. We excluded a comprehensive ELISA-vs-MAT diagnostic accuracy analysis from this paper, preserving the paper’s focus strictly on seroepidemiology.

Although the target sample size was calculated to ensure adequate precision for overall seroprevalence and primary multivariable modeling, subgroups with few observed events risked yielding statistically unstable estimates. To avoid further sparsification of data and generation of excessively wide intervals, subgroup-specific findings were interpreted with caution, and only exposure variables with p < 0.200 in the univariable analysis – and those justified *a priori* – were included in the multivariable model. To assess the robustness of variable selection, five multivariable models were developed; bidirectional selection, full model, backward elimination, backward elimination with sex, settlement, and level of education forced into a model *a priori* as sociodemographic confounders, and least absolute shrinkage and selection operator (LASSO) regression with variable selection informed by 10-fold cross-validation, followed by refitting of LASSO-selected variables as a standard unpenalized logistic regression model. Model fit among the five models was compared using the Akaike Information Criterion (AIC). The strength of association for each covariate was calculated and expressed as adjusted ORs with 95% CI. Variables with p < 0.05 were considered significant and an independent predictor of the outcome variable.

As we sampled participants from households nested within six study communities using probability-proportional-to-size sampling, with limited household-level substitution for non-response (5 households, 1.0%), observations may not be fully independent and intra-cluster correlation could cause conventional logistic regression to underestimate standard errors. This clustering was anticipated at the design stage and therefore we applied a design effect of 2.0 while calculating sample size. However, at the analysis stage, we did not pursue cluster-robust standard errors or a random-intercept mixed-effects logistic model because only six upper-level clusters were available, which is too few to estimate between-cluster variance or ensure model convergence [17,18]. The primary multivariable model therefore uses conventional maximum-likelihood standard errors.

Model diagnostics were conducted for the final multivariable logistic regression model. Multicollinearity among explanatory variables was assessed using variance inflation factors (VIF), with generalized VIF (GVIF) values adjusted for degrees of freedom calculated for categorical predictors with more than two levels. Model calibration was evaluated using the Hosmer–Lemeshow goodness-of-fit test, while discriminatory ability was assessed using the area under the receiver operating characteristic curve (AUC). Influential observations were examined using standardized residuals and Cook’s distance (Cook’s distance > 4/n), and a sensitivity analysis was performed by refitting the final model after excluding these observations. The geographic distribution of seropositive households was mapped using ArcGIS 10.8 (Environmental Systems Research Institute, Inc., Redlands, CA, USA). We performed all statistical analysis in R 4.5.1 (The R Project for Statistical Computing, https://www.r-project.org/).

## Results

### Participant characteristics

Among 503 participants, 11 were excluded for hemolysis or inadequate blood samples. Out of 492 participants, 50.6% (249) were males (Table 2). The mean age of participants was 41.4 years (SD = 13.4, 95% CI: 40.2 – 42.5). Nearly half (47.0%) of the participants lived in urban areas, 13.2% in crowded urban areas and 39.8% in rural areas. Although 32.9% could not read and write, 37.8% had completed secondary education. Occupations varied: while 28.7% were farmers, 24.2% were office-goers and 19.3% were in business. At the time of the study, acute symptoms were infrequent: fever (2.0%), headache (5.7%), myalgia (3.0%) and arthralgia (3.3%), while gastrointestinal symptoms and jaundice were rarer (≤ 1.4%). Within the 12 months prior to the study, 57.1% of participants had sustained skin injuries and 61.2% had experienced fever, although only 4.1% had required hospitalization for fever.

**Table 2:** Sociodemographic characteristics, current symptoms of leptospirosis, skin injuries and fever in the last 12 months among the participants (N = 492).

Variables	n (%)
**Sociodemographic characteristics**	
Age (years)	
18 – 30	101 (20.5)
31 – 40	165 (33.5)
41 – 50	116 (23.6)
51+	110 (22.4)
Sex	
Female	243 (49.4)
Male	249 (50.6)
Level of education	
Cannot read and write	162 (32.9)
Primary (up to Class 6) & monastic education	76 (15.4)
Secondary (Class 7–12)	186 (37.8)
Higher education (Diploma and above)	68 (13.8)
Occupation	
Business	95 (19.3)
Farmer	141 (28.7)
Office-goers	119 (24.2)
Others	137 (27.8)
Settlement	
Urban	231 (47.0)
Crowded urban area	65 (13.2)
Rural	196 (39.8)
Skin injuries in last 12 months	
Yes	281 (57.1)
No	211 (42.9)
Fever in last 12 months	
Yes	301 (61.2)
No	191 (38.8)
Hospitalized due to fever in last 12 months	
Yes	20 (4.1)
No	472 (95.9)
**Current symptoms**	
Fever	
Yes	10 (2.0)
No	482 (98.0)
Headache	
Yes	28 (5.7)
No	464 (94.3)
Myalgia	
Yes	15 (3.0)
No	477 (97.0)
Arthralgia	
Yes	16 (3.3)
No	476 (96.7)
Vomiting	
Yes	3 (0.6)
No	489 (99.4)
Diarrhea	
Yes	1 (0.2)
No	491 (99.8)
Stomachache	
Yes	7 (1.4)
No	485 (98.6)
Jaundice	
Yes	3 (0.6)
No	489 (99.4)

### Seroprevalence and primary reacting serovars

The overall seroprevalence of *Leptospira* spp. based on ELISA IgM was 22.4% (95% CI: 18.8 – 26.4) and, by ELISA IgG, 16.1% (95% CI: 13.0 – 19.7), while using MAT as the reference test against 13 serovars, the seroprevalence was 19.3% (95% CI: 16.0 – 23.1) (Fig 3). As shown in Fig 4, the serogroups (sg)/serovars (sv) reacting in the MAT were sg/sv Australis 49% (47), sg Icterohemorrhagiae sv Lai-like 13% (12), sg Semaranga sv Patoc 10% (10), sg/sv Icterohemorrhagiae 10% (9), sg/sv Grippotyphosa 10% (9), sg/sv Canicola 3% (3), sg/sv Pomona 3% (3), sg Autumnalis sv Bangkinang 1% (1) and sg Sejroe sv Hardjo 1% (1).

**Fig 3:**
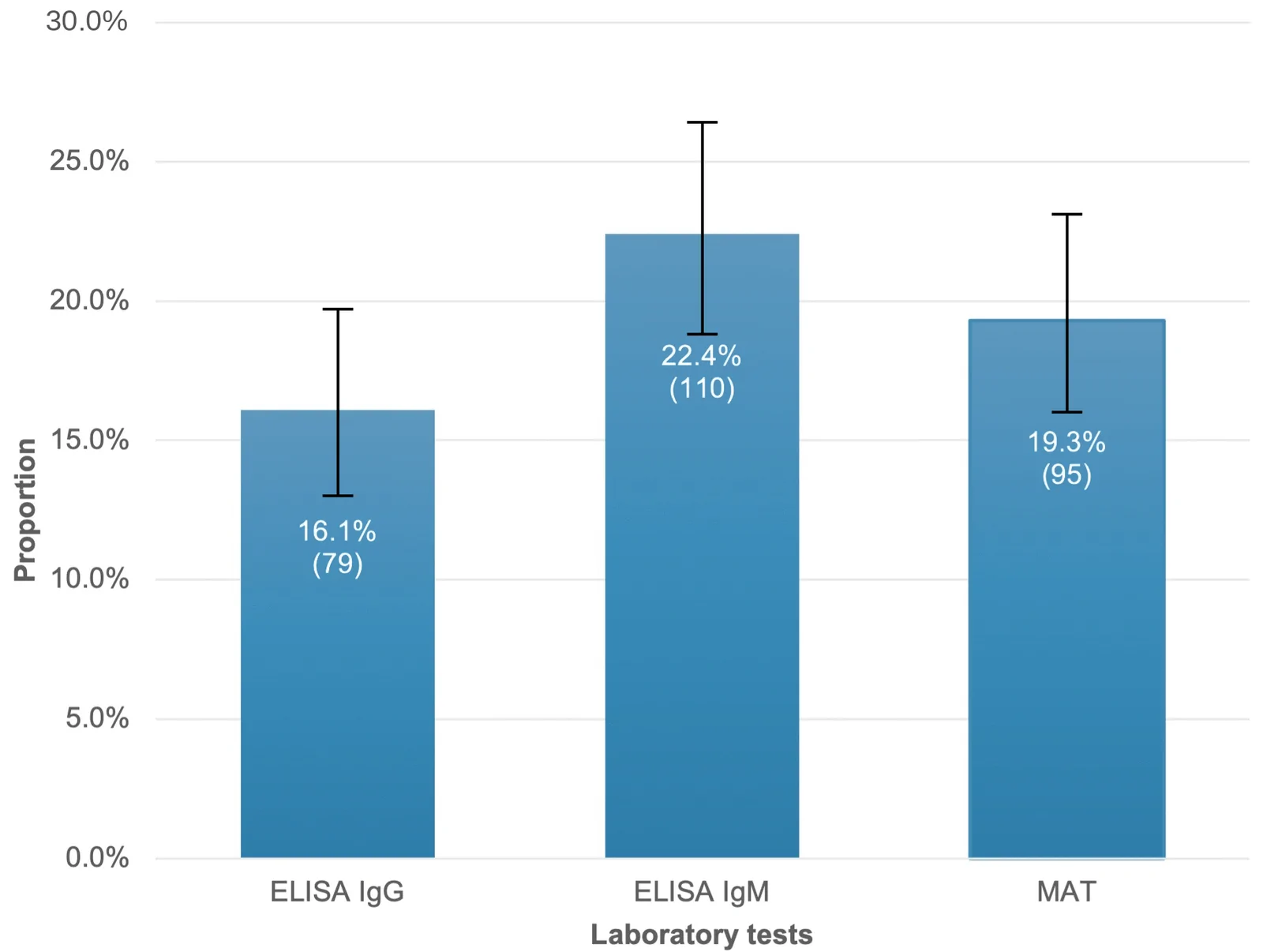
Seroprevalence of *Leptospira* spp. among 492 people in Sarpang, Bhutan, 2024.

**Fig 4:**
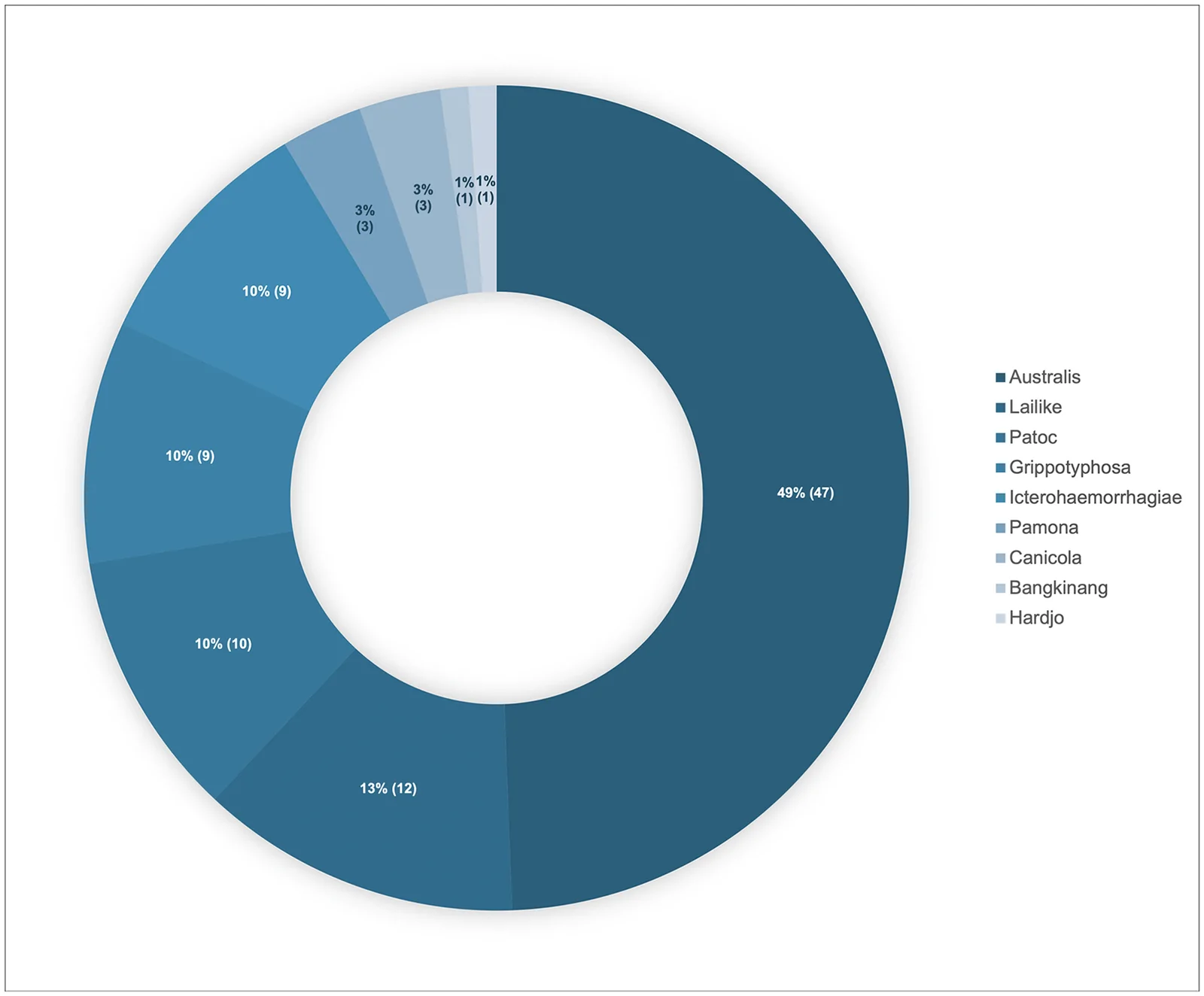
Proportional distribution of *Leptospira* serovars among 95 seropositive participants in Sarpang, Bhutan, 2024.

### Geospatial distribution of *Leptospira* seropositive cases

*Australis* was the most prevalent and widespread serovar, detected in all six study areas – Chhudzom (Fig 5A), Jigme Chhoeling (Fig 5B), Senggye (Fig 5C), Shompangkha (Fig 5D), Gelephu Thromde (Fig 5E) and Umling (Fig 5F) – with the highest MAT seropositivity in Chhudzom (13.6%) and Gelephu Thromde (12.7%), followed by P*atoc,* which was also present in all six study areas, with prevalence rates ranging from 0.8% to 5.7% (Fig 5). Gelephu Thromde had the highest serovar diversity, with nine different serovars detected (Fig 5E). Serovars such as *Grippotyphosa, Lai-like,* and *Icterohemorrhagiae* were observed in multiple locations but at lower prevalence rates.

**Fig 5:**
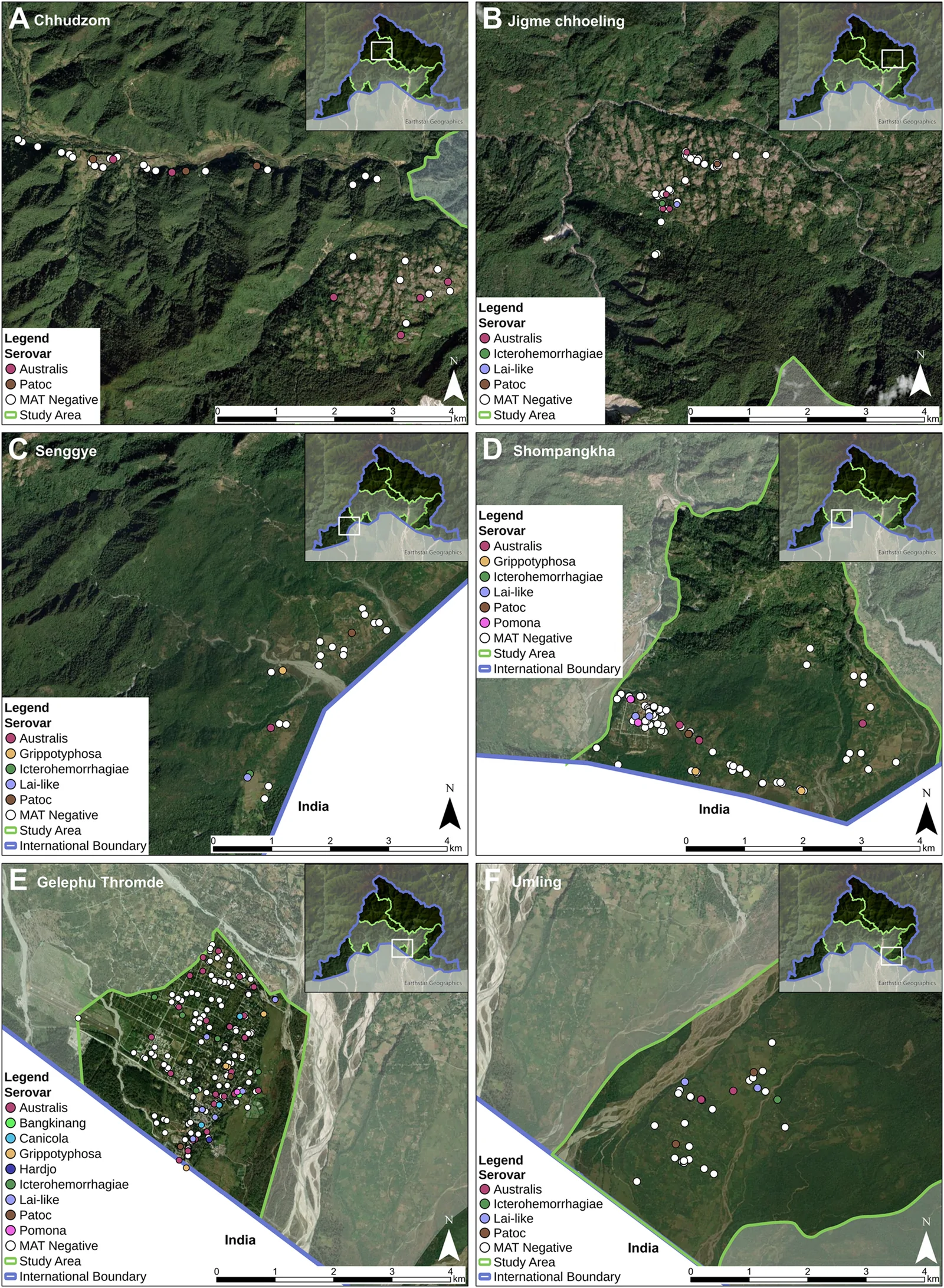
Geospatial distribution of *Leptospira* seropositive cases in (A) Chhudzom, (B) Jigme Chhoeling, (C) Senggye, (D) Shompangkha, (E) Gelephu Thromde and (F) Umling, 2024. The basemap used for geographic visualization is the Esri ArcGIS 10.8 basemap (World Imagery) used under written permission from Esri (Environmental Systems Research Institute, Inc.) granted on 29 July 2026. Content is the intellectual property of Esri and is used herein with permission. Copyright 2026 Esri and its licensors. All rights reserved. Boundary shapefiles for Bhutan were obtained from the Bhutan National Spatial Data Infrastructure (NSDI) System (https://nsdi.systems.gov.bt/), which are publicly available and in the public domain. The geolocation of the study population and seropositive participants was generated and plotted by the authors using geographic information system (GIS) tools in ArcGIS 10.8.

### Exposure to potential behavioral and environmental risk factors

Regarding animal exposure, 61.4% of participants reported contact with one or more animals in the past 12 months (Table 3). Environmental exposures were common: nearly half (48.8%) reported frequent consumption of unboiled water, 43.1% had come into contact with stagnant water and 25.0% had participated in paddy plantation within the 12 months preceding the study. In addition, 6.1% of participants reported exposure to floods, and 28.0% reported walking barefoot.

**Table 3:** Exposure to potential risk factors related to *Leptospira* spp. seropositivity (N = 492).

Risk factors	Variables	n (%)
Animal contacts*	Contact with 1 animal	118 (24.0)
	Contact with > 1 animal	184 (37.4)
	No contact with animals	190 (38.6)
Environmental risk factors*	Is there a garbage facility in the vicinity?	
	Yes	202 (41.1)
	No	290 (58.9)
	Is there an open defecation area in the vicinity?	
	Yes	26 (5.3)
	No	466 (94.7)
	Is there a bat-infested cave in the vicinity?	
	Yes	15 (3.0)
	No	477 (97.0)
	Is there marshy land in the vicinity?	
	Yes	105 (21.3)
	No	387 (78.7)
	Have you come in contact with stagnant water?	
	Yes	212 (43.1)
	No	280 (56.9)
	Have you been exposed to floods?	
	Yes	30 (6.1)
	No	462 (93.9)
Behavioral or occupational risk factors*	Have you participated in paddy plantation?	
	Yes	123 (25.0)
	No	369 (75.0)
	Is there fishery farming in your vicinity?	
	Yes	16 (3.3)
	No	476 (96.7)
	Did you drink water from a pond?	
	Yes	23 (4.7)
	No	469 (95.3)
	Did you drink water from a lake?	
	Yes	7 (1.4)
	No	485 (98.6)
	Do you often drink unboiled water?	
	Yes	240 (48.8)
	No	252 (51.2)
	Did you visit a bat-infested cave?	
	Yes	9 (1.8)
	No	483 (98.2)
	Do you walk barefoot?	
	Yes	138 (28.0)
	No	354 (72.0)
	Do you often visit the forest?	
	Yes	208 (42.3)
	No	284 (57.7)
	Have you come in contact with dead mammals?	
	Yes	55 (11.2)
	No	437 (88.8)
Recreational activities*	Did you bathe or swim in the river?	
	Yes	118 (24.0)
	No	374 (76.0)
	Did you bathe or swim in the pond?	
	Yes	40 (8.1)
	No	452 (91.9)
	Did you bathe or swim in the lake?	
	Yes	4 (0.8)
	No	488 (99.2)

*All re-call periods of exposure were within the 12 months preceding the study.

Environmental conditions near residences included proximity to garbage disposal sites (41.1%), marshy land (21.3%) and open defecation areas (5.3%). Fish farming and presence of bat-infested caves in the vicinity were reported by 3.3% and 3.0% of participants, respectively. Direct exposure to natural water bodies was also noted, with 24.0% of the participants having bathed or swum in rivers, 8.1% in ponds, and 0.8% in lakes within the 12 months preceding the study. Furthermore, 42.3% reported regular forest visits, 11.2% had come into contact with dead mammals, and 1.8% had entered bat-infested caves.

### Potential risk factors for MAT seropositivity

The univariable analysis identified several factors significantly associated with MAT seropositivity. Participation in paddy plantation showed the strongest positive association (OR: 3.09; 95% CI: 1.93 – 4.96; p < 0.001), followed by contact with dead mammals (OR: 2.27; 95% CI: 1.23 – 4.21; p = 0.009), drinking unboiled water (OR: 2.19; 95% CI: 1.37 – 3.47; p = 0.001), contact with stagnant water (OR: 1.70; 95% CI: 1.08 – 2.67; p = 0.021) and male sex (OR: 1.89; 95% CI: 1.19 – 2.99; p = 0.007). Relative to rural residents, those living in a crowded urban area were associated with higher odds of MAT seropositivity (OR: 1.96; 95% CI: 1.01 – 3.81; p = 0.046). Likewise, participants with primary education (OR: 2.75; 95% CI: 1.43 – 5.30; p = 0.002) and unable to read and write (OR: 1.93; 95% CI: 1.09 – 3.40; p = 0.023) relative to secondary education were associated with higher odds of MAT seropositivity.

Ten variables meeting the p < 0.200 threshold in univariable analysis were carried forward to multivariable models (Table 4). Five models yielded closely comparable model fit (AIC 433.96 – 437.79), indicating that model fit did not depend materially on the variable-selection algorithm used. The model incorporating sex, settlement, and level of education as forced sociodemographic covariates (AIC = 434.24) was adopted as the final model, since these variables are established *a priori* confounders in leptospirosis seroepidemiology and their inclusion did not meaningfully compromise model fit relative to the minimum-AIC alternative (ΔAIC = 0.28). Overall, the final sample of 492 participants (including 95 MAT-positive cases) provided a reliable basis to estimate seroprevalence and build the primary multivariable model; however, individual subgroups with few observed events (e.g., hospitalization for fever, with only one MAT-positive case) required cautious interpretation.

**Table 4:** Crude and adjusted odds ratios (ORs) for potential risk factors associated with *Leptospira* spp. seropositivity in MAT (N = 492).

Variables*	MAT Positive n (%)	Unadjusted analysis		Adjusted analysis	
		OR (95% CI)	P-value	Adjusted OR (95% CI)	P-value
Settlement					
Rural	32 (16.3)	Reference	–	Reference	–
Crowded urban area	18 (27.7)	1.96 (1.01 – 3.81)	0.046	4.08 (1.83 – 9.13)	**0.001**
Urban	45 (19.5)	1.24 (0.75 – 2.04)	0.399	2.94 (1.46 – 5.91)	**0.002**
Sex					
Female	35 (14.4)	Reference	–	Reference	–
Male	60 (24.1)	1.89 (1.19 – 2.99)	0.007	1.43 (0.84 – 2.44)	0.191
Level of education					
Cannot read and write	36 (22.2)	1.93 (1.09 – 3.40)	0.023	2.02 (1.06 – 3.85)	**0.034**
Primary (up to Class 6) & monastic education	22 (28.9)	2.75 (1.43 – 5.30)	0.002	2.52 (1.21 – 5.24)	**0.013**
Secondary (Class 7–12)	24 (12.9)	Reference	–	Reference	**–**
Higher education	13 (19.1)	1.60 (0.76 – 3.35)	0.217	1.72 (0.76 – 3.90)	0.192
Skin injuries in last 12 months					
No	33 (15.6)	Reference	–		
Yes	62 (22.1)	1.53 (0.96 – 2.43)	0.075		
Fever in last 12 months					
No	43 (22.5)	Reference	–	Reference	–
Yes	52 (17.3)	0.72 (0.46 – 1.13)	0.152	0.39 (0.22 – 0.69)	**0.001**
Hospitalized for fever in last 12 months					
No	94 (19.9)	Reference	–	Reference	–
Yes	1 (5.0)	0.21 (0.03 – 1.60)	0.133	0.18 (0.02 – 1.41)	0.102
Stagnant water contact in last 12 months					
No	44 (15.7)	Reference	–	Reference	–
Yes	51 (24.1)	1.70 (1.08 – 2.67)	0.021	1.68 (0.99 – 2.87)	0.056
Participated in paddy plantation in last 12 months					
No	53 (14.4)	Reference	–	Reference	–
Yes	42 (34.1)	3.09 (1.93 – 4.96)	< 0.001	4.74 (2.49 – 9.01)	**< 0.001**
Drink unboiled water in last 12 months					
No	34 (13.5)	Reference	–	Reference	–
Yes	61 (25.4)	2.19 (1.37 – 3.47)	0.001	2.34 (1.35 – 4.06)	**0.002**
Contact with dead mammals in last 12 months					
No	77 (17.6)	Reference	–		
Yes	18 (32.7)	2.27 (1.23 – 4.21)	0.009		

*Only variables with p < 0.200 during univariable analysis are presented in this table. For categorical variables, the reference category is indicated (“Reference”); for binary exposure variables, “No” is the reference category. “–”, not applicable (reference category).

During the multivariable logistic regression, participation in paddy plantation (adjusted OR: 4.74; 95% CI: 2.49 – 9.01; p < 0.001) and drinking unboiled water (adjusted OR: 2.34; 95% CI: 1.35 – 4.06; p = 0.002) were associated with increased odds of MAT seropositivity (Table 4; S1 Table). Contact with stagnant water showed a borderline association (adjusted OR: 1.68; 95% CI: 0.99 – 2.87; p = 0.056). Relative to rural residents, participants in crowded urban areas (adjusted OR: 4.08; 95% CI: 1.83 – 9.13; p = 0.001) and urban areas (adjusted OR: 2.94; 95% CI: 1.46 – 5.91; p = 0.002) had significantly increased odds of MAT seropositivity. Relative to participants with secondary education, odds were significantly higher for those with primary education (adjusted OR: 2.52; 95% CI: 1.21 – 5.24; p = 0.013) and those who could not read and write (adjusted OR: 2.02; 95% CI: 1.06 – 3.85; p = 0.034). On the other hand, fever in the past 12 months was associated with reduced odds of seropositivity (adjusted OR: 0.39; 95% CI: 0.22 – 0.69; p = 0.001).

Multicollinearity was assessed for the final model. All variance inflation factors were low (GVIF^(1/(2 × Df)) range: 1.01 – 1.26), indicating no evidence of problematic collinearity among predictors. The Hosmer–Lemeshow test indicated adequate model calibration (χ^2^ = 8.46, df = 8, p = 0.390). The model demonstrated acceptable discriminatory ability, with an AUC of 0.765 (95% CI: 0.714 – 0.816). Based on Cook’s distance, 39 observations (7.9%) were flagged as potentially influential. Sensitivity analysis excluding these observations altered several coefficient estimates; notably, the odds ratio for hospitalization for fever became inestimable, as the single MAT seropositive case within this exposure category (1 of 20 hospitalized individuals) was among the observations excluded. The odds ratio for paddy plantation participation also increased substantially from 4.74 (95% CI: 2.49 – 9.01) to 41.91 (95% CI: 12.90 – 136.18). Examination of the excluded observations showed that they were disproportionately drawn from MAT seropositive participants, reducing the number of positive outcomes available to the model from 95 to approximately 63 (a 34% reduction), suggesting that the standard influence diagnostics preferentially flagged observations from the minority (seropositive) outcome category. However, the large change in the paddy plantation estimate indicates that this estimate is sensitive to a subset of observations and should be interpreted with caution. Given this, estimates from the original model retaining all 492 observations are reported as the primary analysis.

## Discussion

This study found one in five participants seropositive for anti-*Leptospira* antibodies in a subtropical district in Bhutan, providing empirical support for our hypothesis that environmental and occupational contact with potentially contaminated water or soil would be associated with seropositivity. While Australis and Lai-like were the predominant circulating serovars, Australis and Patoc had the most extensive geographical distribution. In the final multivariable model, paddy farming and drinking unboiled water – environmental and occupational exposures as hypothesized – were associated with MAT seropositivity, whereas contact with stagnant water showed only a borderline association (adjusted OR: 1.68; 95% CI: 0.99 – 2.87; p = 0.056).

Our study identified a seropositivity of 19.3% by MAT, a figure substantially higher than the 1.6% MAT-based seroprevalence reported in the only previous seroprevalence study in Bhutan [13]. The marked difference is likely due to seasonal and methodological variations, environmental exposure and geographical settings; for example, the previous study performed MAT on only 45% of the total samples, whereas our study performed MAT on all samples. In contrast, the ELISA-based estimates were similar in the two studies (IgG 16.1% and IgM 22.4% here versus 17.6% previously), suggesting that the difference lies mainly in the confirmatory MAT results. The previous study [13] found a large gap between ELISA and MAT seropositivity (17.6% versus 1.6%), which we did not observe. In addition, interpretation of seroprevalence estimates should consider the MAT seropositivity threshold used. We adopted a cutoff titer of ≥ 1:80 to identify serological evidence of previous exposure rather than acute infection, which may yield higher seroprevalence estimates than studies using more stringent cutoffs (e.g., ≥ 1:160 or ≥ 1:400); therefore, direct comparison across studies should be interpreted cautiously. Meanwhile, previous studies used varying cutoff titers to determine seroprevalence, ranging from 1:40–1:100 [19–24].

Across Asia, leptospirosis shows considerable heterogeneity. India reported 9.47% seroprevalence, with higher prevalence rates in coastal states [25]; Malaysia recorded 1,976 cases/69 deaths in 2010 and 5,284 cases/52 deaths in 2016 [26,27]; in Nepal, 21% of febrile Terai patients tested IgM-positive [28]; seropositivity reached 38% in a flood-prone district of Bangladesh, and 18% of dengue-negative patients in Dhaka in 2000 had evidence of infection [5,29]; Sri Lanka had over 7,000 cases in 2008 [26,30]; and in Thailand, young males showed 28% seroprevalence, ranging from 10% to 52% by province [31]. These patterns highlight that leptospirosis remains a widespread yet under-recognized public health problem in Asia, providing a relevant context for interpreting our findings from Bhutan.

Residents of crowded urban areas with small houses, and to a lesser extent urban residents, had significantly higher adjusted odds of MAT seropositivity than rural residents (adjusted OR 4.08 and 2.94, respectively). Notably, the urban-rural contrast was weaker before adjustment (crude OR for urban residents 1.24; 95% CI: 0.75 – 2.04), which may indicate negative confounding, for example by paddy plantation and other agricultural exposures that are more common among rural residents. The lower odds among rural residents should therefore be interpreted cautiously. The higher seropositivity among residents of crowded urban areas with small houses may reflect environmental conditions that are often associated with low-income urban settings, including substandard housing, inadequate sanitation and increased opportunities for contact with animal reservoirs [32]. Education was also associated with MAT seropositivity; relative to participants with secondary education, odds of seropositivity were significantly higher among those with primary and those unable to read and write. This association may reflect differences in occupational exposure and awareness of preventive measures among individuals with lower educational attainment, although these factors were not directly assessed in the present study.

We identified the strongest association between participating in paddy farming and seropositivity. During the rainy season, rural communities engage in paddy plantation, and some urban residents travel to ancestral villages to help their relatives with the work. Paddy plantation was also identified as a major risk factor in India [33], Japan [34] and Sri Lanka [35]. In Bhutan, traditional rice-farming practices involve inundating rice terraces and keeping them flooded for several days or weeks to allow organic matter to decay into compost manure and to control weeds. Such prolonged stagnation of water creates ideal environmental conditions for the persistence of *Leptospira* introduced through the urine of infected animal reservoirs. Contaminated soil and water serve as important environmental sources of human exposure, particularly during the periods of heavy rainfall, flooding and peak agricultural seasons [36–39]. These findings support targeted preventive measures for high-risk individuals in subtropical Bhutan.

Drinking unboiled water was also associated with seropositivity, consistent with a finding in Vietnam [40]. Drinking unboiled water likely reflects consumption of untreated water from taps or natural water sources. Heavy rainfall likely facilitates dispersion of *Leptospira* from soil to surface water and drinking water sources [38]. Although oral transmission of leptospirosis is unlikely due to the protection from saliva, intact oral mucosa and gastric juice [41], past exposure through broken skin or mucous membranes while fetching or drinking untreated water is plausible. In contrast to previous studies [39,42–44], recreational water activities were not associated with seropositivity in this study. This may partly reflect the small numbers of participants who swam or bathed in ponds (8.1%) and lakes (0.8%), although river bathing was more common (24%). In addition, the lack of association with river-related recreational activities may partly reflect the predominance of fast-flowing rivers in Bhutan, where dilution, turbulent flow, and shorter duration of water contact may reduce the environmental persistence of *Leptospira* and opportunities for human exposure.

An unexpected finding was that participants reporting fever in the preceding 12 months had lower adjusted odds of MAT seropositivity. This inverse association should be interpreted cautiously, as MAT primarily reflects previous exposure rather than recent infection, whereas fever was assessed only for the preceding 12 months. The finding may therefore reflect temporal mismatch between exposure and outcome ascertainment, recall bias, or residual confounding rather than a true protective association.

Our study has several important limitations. First, due to limited resources, we excluded the animal population in our serological testing, which restricts our understanding of transmission patterns. Nonetheless, a seroprevalence study such as this one cannot determine transmission pathways, which warrants molecular studies in the future. Second, the use of the MAT as a reference test has several shortcomings, including delayed seroconversion and variability in titer readings among laboratory scientists, often by one titer cutoff [7,13]. Furthermore, our panel of 13 serovars was based on global prevalence and may not fully reflect the epidemiological reality of Bhutan and potentially underestimate true local seroprevalence. Conversely, the panel included a saprophytic strain (*L. biflexa* strain Patoc I), which cross-reacts with human antibodies generated by a number of pathogenic serovars [7]. The use of PCR-based molecular methods as a complementary test to MAT and ELISA would have captured some acute leptospirosis infections. Third, while our sample was representative of the population of the district, the findings may not be generalizable to the entire country, as circulating serovars and climatic conditions vary across the country [45]. In addition, the exclusion of non-Bhutanese might have excluded foreign laborers and migrant workers, with distinctly different occupational exposure profiles. Fourth, the use of a 12-month recall period may have introduced recall bias and exposure misclassification. In addition, some exposure variables were assessed without detailed contextual information (e.g., source of drinking water), which may have limited the interpretation of observed associations. Fifth, although model diagnostics indicated adequate calibration and no evidence of problematic multicollinearity, sensitivity analysis revealed unstable estimates for exposures with few observed events, notably hospitalization for fever, for which only one MAT seropositive case was recorded. This instability reflects, at least in part, the limited number of events within these subgroups rather than a weakness of the final model itself, and estimates for such variables should therefore be interpreted with caution pending confirmation in larger studies. Sixth, although a design effect was incorporated at the sample-size stage, the small number of primary sampling units (six communities) meant that neither a random-effects mixed model nor cluster-robust standard errors could be reliably estimated. Therefore, we report conventional, non-cluster-adjusted standard errors for the primary multivariable model; and confidence intervals may be modestly underestimated for community-linked exposures such as paddy farming participation and the findings should be interpreted cautiously. We suggest the need for cluster-robust sensitivity analyses and larger, multi-site studies to confirm the stability of the reported associations.

Despite these limitations, our study provides critical insights for One Health interventions and evidence-based public health decisions. We recommend educating communities at risk about preventive behaviors, including the promotion of protective clothing, such as safety boots and gloves, which may help reduce occupational exposure to the bacteria [39]. We also recommend equipping regional hospitals with diagnostic facilities for laboratory-based surveillance and testing of undifferentiated febrile cases for leptospirosis. Early diagnosis is vital; laboratory testing not only helps confirm diagnosis and identify serovars causing the infection, but also informs surveillance for effective control strategies [7]. Also, early diagnosis and intervention are important, because the acute phase of leptospirosis is responsive to low-cost antibiotics such as benzyl penicillin, doxycycline, cephalosporins and macrolides and most patients recover without complications [4,39]. In contrast, once the disease progresses to the severe immune phase with multiple organ failure, mortality is considerably higher (about 52% in some intensive-care series) and clinical benefit becomes less certain [3,46]. The World Health Organization also recommends initiating antimicrobial therapy before the fifth day of disease onset [7], underscoring the importance of early interventions. In addition, hydrological impacts of projected climate change are expected to increase human exposure to leptospirosis [12], and lower-income countries, which often lack adequate preparedness, timely diagnosis and treatment, and infrastructure, will bear the disproportionate burden of increased incidence [12,47].

The One Health [48] approach in recent years successfully advocated for a comprehensive and multi-disciplinary approach in dealing with diseases of epidemic or pandemic potential [49]. Although leptospirosis is an emblematic One Health disease considering its complex transmission cycles, piecemeal resources allocated for this disease have sustained its vicious cycle of neglect [49]. Inadequacy of public health intervention in Bhutan is such that, although the disease was designated a notifiable disease between 2008 and 2018, the lack of hospital diagnostics and underreporting by healthcare professionals – despite the requirement to report – impeded systematic surveillance and true reporting [50]. The reasons for its removal from the list of notifiable disease are unclear but may reflect an assumption of low disease burden, whereas the true burden of leptospirosis was probably underestimated. Bhutan should therefore improve hospital diagnostics to build a resilient healthcare system and enhance research capacity for leptospirosis management.

## Conclusion

In conclusion, serological evidence of exposure to *Leptospira* spp. was identified among residents of subtropical Bhutan, with serological reactivity to a range of serovars and environmental conditions that may facilitate human exposure. These findings point to actionable public health interventions: (1) educate communities on preventive behaviors, (2) promote use of protective gear during high-risk activities, (3) expand research and surveillance capacity to better understand the epidemiology of leptospirosis, and (4) strengthen hospital-based diagnostics to enable early treatment and reduce complications from leptospirosis in Bhutan. This study provides baseline information on seroprevalence of *Leptospira* exposure for future molecular investigations, including studies of acute illness, to better understand transmission pathways and guide targeted public health interventions.

## Supporting information

S1 Table Crude and adjusted odds ratios (ORs) for potential risk factors associated with MAT seropositivity (N = 492) in detail. (DOCX)

S2 Table Data collection pro forma for “Seroepidemiology of human leptospirosis in subtropical Bhutan, 2024”. (DOCX)

S1 Data Anonymized dataset used for analysis. (XLSX)
