# Supplementary material for: Seroepidemiology of human leptospirosis in subtropical Bhutan, 2024
Source: PLoS Negl Trop Dis. 2026 Nov 4;20(11):e0014821. doi: 10.1371/journal.pntd.0014821 (PMC13734453; doi:10.1371/journal.pntd.0014821)
Supplement: Supplementary file 1 [file pntd.0014821.s001.docx]

**S1 Table. Crude and adjusted odds ratio (OR) for potential risk factors associated with *Leptospira* spp. MAT seropositivity (N = 492)**

| **Sociodemographic characteristics** |  | **MAT Positive n (%)** | **Crude OR (95% CI)** | **P-value** | **Adjusted OR (95% CI)** | **P-value** |
| --- | --- | --- | --- | --- | --- | --- |
| Age Group | 18–30 | 18 (17.8) | Reference | – |  |  |
|  | 31–40 | 33 (20.0) | 1.15 (0.61 – 2.18) | 0.662 |  |  |
|  | 41–50 | 20 (17.2) | 0.96 (0.48 – 1.94) | 0.911 |  |  |
|  | 51+ | 24 (21.8) | 1.29 (0.65 – 2.54) | 0.468 |  |  |
| Settlement | Rural | 32 (16.3) | Reference | – | Reference | -- |
|  | Crowded urban area | 18 (27.7) | 1.96 (1.01 – 3.81) | 0.046 | 4.08 (1.83 – 9.13) | **0.001** |
|  | Urban | 45 (19.5) | 1.24 (0.75 – 2.04) | 0.399 | 2.94 (1.46 – 5.91) | **0.002** |
| Sex | Female | 35 (14.4) | Reference | – | Reference | -- |
|  | Male | 60 (24.1) | 1.89 (1.19 – 2.99) | 0.007 | 1.43 (0.84 – 2.44) | 0.191 |
| Education Level | Cannot read and write | 36 (22.2) | 1.93 (1.09 – 3.40) | 0.023 | 2.02 (1.06 – 3.85) | **0.034** |
|  | Primary (up to class 6) & monastic education | 22 (28.9) | 2.75 (1.43 – 5.30) | 0.002 | 2.52 (1.21 – 5.24) | **0.013** |
|  | Secondary (Class 7 to 12) | 24 (12.9) | Reference | – | Reference | -- |
|  | Higher Education | 13 (19.1) | 1.60 (0.76 – 3.35) | 0.217 | 1.72 (0.76 – 3.90) | 0.192 |
| Occupation | Business | 14 (14.7) | Reference | – |  |  |
|  | Farmer | 29 (20.6) | 1.50 (0.74 – 3.01) | 0.257 |  |  |
|  | Office-goers | 25 (21.0) | 1.54 (0.75 – 3.16) | 0.240 |  |  |
|  | Others | 27 (19.7) | 1.42 (0.70 – 2.88) | 0.330 |  |  |
| Skin injuries in past 12 months | No | 33 (15.6) | Reference | – |  |  |
|  | Yes | 62 (22.1) | 1.53 (0.96 – 2.43) | 0.075 |  |  |
| Fever in past 12 months | No | 43 (22.5) | Reference | – | Reference | -- |
|  | Yes | 52 (17.3) | 0.72 (0.46 – 1.13) | 0.152 | 0.39 (0.22 – 0.69) | **0.001** |
| Hospitalized for fever in past 12 months | No | 94 (19.9) | Reference | – | Reference | -- |
|  | Yes | 1 (5.0) | 0.21 (0.03 – 1.60) | 0.133 | 0.18 (0.02 – 1.41) | 0.102 |
| **Current symptoms** |  |  |  |  |  |  |
| Fever | No | 95 (19.7) | Reference | – |  |  |
|  | Yes | 0 (0.0) | NE | NE |  |  |
| Headache | No | 91 (19.6) | Reference | – |  |  |
|  | Yes | 4 (14.3) | 0.68 (0.23 – 2.02) | 0.490 |  |  |
| Myalgia | No | 95 (19.9) | Reference | – |  |  |
|  | Yes | 0 (0.0) | NE | NE |  |  |
| Arthralgia | No | 92 (19.3) | Reference | – |  |  |
|  | Yes | 3 (18.8) | 0.96 (0.27 – 3.45) | 0.954 |  |  |
| Vomiting | No | 94 (19.2) | Reference | – |  |  |
|  | Yes | 1 (33.3) | 2.10 (0.19 – 23.42) | 0.546 |  |  |
| Diarrhea | No | 95 (19.3) | Reference | – |  |  |
|  | Yes | 0 (0.0) | NE | NE |  |  |
| Stomachache | No | 94 (19.4) | Reference | – |  |  |
|  | Yes | 1 (14.3) | 0.69 (0.08 – 5.83) | 0.736 |  |  |
| Jaundice | No | 95 (19.4) | Reference | – |  |  |
|  | Yes | 0 (0.0) | NE | NE |  |  |
| **Animal contact*** | | | | | | |
| Contact with animals | No | 42 (22.1) | Reference | **–** |  |  |
|  | 1 animal | 22 (18.6) | 0.81 (0.45 – 1.44) | 0.467 |  |  |
|  | > 1 animal | 31 (16.8) | 0.71 (0.43 – 1.20) | 0.201 |  |  |
| **Environmental Risk Factors*** | | | | | | |
| Garbage facilities in the vicinity | No | 52 (17.9) | Reference | – |  |  |
|  | Yes | 43 (21.3) | 1.24 (0.79 – 1.94) | 0.354 |  |  |
| Open defecation in the vicinity | No | 91 (19.5) | Reference | – |  |  |
|  | Yes | 4 (15.4) | 0.75 (0.25 – 2.23) | 0.604 |  |  |
| Bat-infested cave in the vicinity | No | 94 (19.7) | Reference | – |  |  |
|  | Yes | 1 (6.7) | 0.29 (0.04 – 2.24) | 0.236 |  |  |
| Marshy land in the vicinity | No | 74 (19.1) | Reference | – |  |  |
|  | Yes | 21 (20.0) | 1.06 (0.62 – 1.82) | 0.840 |  |  |
| Stagnant water contact | No | 44 (15.7) | Reference | – | Reference | -- |
|  | Yes | 51 (24.1) | 1.70 (1.08 – 2.67) | 0.021 | 1.68 (0.99 – 2.87) | **0.056** |
| Exposure to flood | No | 88 (19.0) | Reference | – |  |  |
|  | Yes | 7 (23.3) | 1.29 (0.54 – 3.11) | 0.565 |  |  |
| **Behavioral or Occupational Risk Factors*** | | | | | | |
| Participated in paddy plantation | No | 53 (14.4) | Reference | – | Reference | -- |
|  | Yes | 42 (34.1) | 3.09 (1.93 – 4.96) | <0.001 | 4.74 (2.49 – 9.01) | **<0.001** |
| Fishery farming in the vicinity | No | 90 (18.9) | Reference | – |  |  |
|  | Yes | 5 (31.3) | 1.95 (0.66 – 5.75) | 0.226 |  |  |
| Drink pond water | No | 89 (19.0) | Reference | – |  |  |
|  | Yes | 6 (26.1) | 1.51 (0.58 – 3.93) | 0.402 |  |  |
| Drink lake water | No | 94 (19.4) | Reference | – |  |  |
|  | Yes | 1 (14.3) | 0.69 (0.08 – 5.83) | 0.736 |  |  |
| Drink unboiled water | No | 34 (13.5) | Reference | – | Reference | -- |
|  | Yes | 61 (25.4) | 2.19 (1.37 – 3.47) | <0.001 | 2.34 (1.35 – 4.06) | **0.002** |
| Visited bat-infested cave | No | 94 (19.5) | Reference | – |  |  |
|  | Yes | 1 (11.1) | 0.52 (0.06 – 4.19) | 0.537 |  |  |
| Walk barefoot | No | 64 (18.1) | Reference | – |  |  |
|  | Yes | 31 (22.5) | 1.31 (0.81 – 2.13) | 0.269 |  |  |
| Visits forest often | No | 52 (18.3) | Reference | – |  |  |
|  | Yes | 43 (20.7) | 1.16 (0.74 – 1.82) | 0.512 |  |  |
| Contact with dead mammals | No | 77 (17.6) | Reference | – |  |  |
|  | Yes | 18 (32.7) | 2.27 (1.23 – 4.21) | 0.009 |  |  |
| **Recreational Activities*** | | | | | | |
| Swimming or bathing in river | No | 73 (19.5) | Reference | – |  |  |
|  | Yes | 22 (18.6) | 0.94 (0.56 – 1.60) | 0.834 |  |  |
| Swimming or bathing in pond | No | 86 (19.0) | Reference | – |  |  |
|  | Yes | 9 (22.5) | 1.24 (0.57 – 2.69) | 0.594 |  |  |
| Swimming or bathing in lake | No | 95 (19.5) | Reference | – |  |  |
|  | Yes | 0 (0.0) | NE | NE |  |  |

OR, odds ratio; CI, confidence interval; MAT, microscopic agglutination test; NE, not estimable (no MAT-positive participants in the category); –, not applicable. n (%) is the number of MAT-positive participants in each category with the row percentage. Crude ORs are from univariable logistic regression. Adjusted ORs are from the final multivariable model (sex, settlement and level of education forced in as sociodemographic covariates); blank adjusted cells indicate variables not included in the final model. *Exposure to the potential risk factors within 12 months preceding the study period. All categories of each categorical variable are shown; the reference category of each variable is labelled “Reference” in the crude column (and in the adjusted column for variables retained in the final model), and for binary variables “No” is the reference category. Reference categories: age 18–30 years; settlement, rural; sex, female; level of education, secondary (Class 7 to 12); occupation, business; contact with animals, none.
