# Supplementary material for: Seroepidemiology of human leptospirosis in subtropical Bhutan, 2024
Source: PLoS Negl Trop Dis. 2026 Nov 4;20(11):e0014821. doi: 10.1371/journal.pntd.0014821 (PMC13734453; doi:10.1371/journal.pntd.0014821)
Supplement: Supplementary file 2 [file pntd.0014821.s002.docx]

**S2 Table. Data collection pro forma for “Sero-epidemiology of human leptospirosis in subtropical Bhutan, 2024”**

| These patient identifiers are collected to avoid duplication of patients and matching it to their serum samples. These identifiers will be deleted after data has been validated and will not be a part of the dataset that will be analysed. | |
| --- | --- |
|  | |
| **Patient’s name** |  |
|  |  |
| **Citizenship number** |  |
|  |  |
| **Phone number** |  |

| **Date** |  |
| --- | --- |
| **Participant’s name** |  |
| **Sample code** | Eg; LB002 |
| **Citizenship Identity Card no.** | 11 digits |
| **Phone no.** |  |
| **Household geolocation (GPS)** |  |
| Age |  |
| Sex | Male  Female |
| Gewog (Blocks) | **[Drop down]**  Senggye  Shompangkha  Gelephu Thromde  Umling  Jigme Chhoeling/Suray  Chhudzom/Doban |
| Chiwog (sub-blocks) |  |
| Level of education | **[Drop down]**  Cannot read and write  Primary (up to Class VI)  Secondary (up to Class XII)  Certificate/Diploma  Degree  Masters and above  Monastic education  Others |
| Occupation | **[Drop down]**  Farmer  Labourer  Government service  Private employee  Military service  Religious figure/Monks/nuns  Business  Retired  Dependent  Students  Fishery farmer  Forester  Local government  Others |
| Settlement | **[Drop down]**  Rural  Urban  Crowded urban area |
| Do you have any of the following symptoms? | **[Multiple check box]**  Fever  Headache  Myalgia (generalized body ache)  Arthralgia (joint pain)  Vomiting  Diarrhea  Abdominal pain  Jaundice  Others  None |
| Domesticated mammals in the vicinity | **[Multiple check box]**  Rat  Rabbit  Bat  Cow  Goat  Cattle  Dog  Cat  Horses  Pig  None |
| Did you have contact with stagnant water in last 12 months? | **[Drop down]**  Yes  No |
| Were you exposed to floods in last 12 months? | **[Drop down]**  Yes  No |
| Did you participate in paddy plantation in last 12 months? | **[Drop down]**  Yes  No |
| Are there garbage collection facility in the locality? | **[Drop down]**  Yes  No |
| Is there open defecation area in your locality? | **[Drop down]**  Yes  No |
| Is there bat-infested cave in the vicinity? | **[Drop down]**  Yes  No |
| Is there fishery farming in your vicinity? | **[Drop down]**  Yes  No |
| Is there marshy land in the vicinity? | **[Drop down]**  Yes  No |
| Did you batch or swim in river in last 12 months? | **[Drop down]**  Yes  No |
| Did you bath or swim in ponds in last 12 months? | **[Drop down]**  Yes  No |
| Did you bath or swim in lake in last 12 months? | **[Drop down]**  Yes  No |
| Did you drink water from pond in last 12 months? | **[Drop down]**  Yes  No |
| Did you drink water from lake in last 12 months? | **[Drop down]**  Yes  No |
| Do you often drink unboiled water? | **[Drop down]**  Yes  No |
| Did you sustain skin injuries in past 12 months? | **[Drop down]**  Yes  No |
| Did you visit bat-infested cave in last 12 months? | **[Drop down]**  Yes  No |
| Do you walk barefoot? | **[Drop down]**  Yes  No |
| Do you often visit forest? | **[Drop down]**  Yes  No |
| Have you come in contact with dead mammals? | **[Drop down]**  Yes  No |
| Did you have fever in past 12 months? | **[Drop down]**  Yes  No |
| Have you got hospitalized due to fever in last 12 months? | **[Drop down]**  Yes  No |
| Blood sample collected | **[Drop down]**  Yes  No |
| Name of sample/data collectors |  |
