# Supplementary material for: Seroepidemiology of human leptospirosis in subtropical Bhutan, 2024
Source: PLoS Negl Trop Dis. 2026 Nov 4;20(11):e0014821. doi: 10.1371/journal.pntd.0014821 (PMC13734453; doi:10.1371/journal.pntd.0014821)
Supplement: Supplementary file 3 [file pntd.0014821.s004.docx]

This manuscript addresses an important and understudied topic, and the seroprevalence estimates and contextual description of exposures are potentially informative for the region. However, there are substantial concerns that undermine confidence in the study's inferential components and require major revision before the work can be considered for publication.

Key issues relate to methodological clarity and analytical justification. The MAT procedures described raise questions about culture conditions, dilution ranges, and consistency of execution, which affect confidence in the reference test. The rationale for conducting ELISA alongside MAT is not carried into the analysis, as only MAT results are used for risk factor modeling without justification. Several exposure definitions lack operational clarity. Interpretation sometimes confuses serological exposure to clinical disease.

**Abstract and introduction**

The background would benefit from clearer framing of the specific research gap being addressed in the context of the Bhutanese population. While the authors mention limited diagnostics as a constraint, it is not clearly explained why human sero-surveillance for leptospirosis in Bhutan is particularly important, nor how diagnostic limitations directly affect this investigation. Additionally, diagnostic test evaluation was not stated as an objective of the study, and no such evaluation appears in the analysis.

It should be clarified that MAT primarily determines **serogroups**, not definitive serovars, due to known cross-reactivities within serogroups.

The manuscript should also clarify whether there were positive participants for both IgM and IgG, how these were handled analytically, and how ELISA results compared with MAT findings.

The statement *“Leptospirosis can be treated with low-cost antibiotics if detected early but efficacy drops once serious complications like jaundice and organ failure develop”* would read more accurately as: *“…but prognosis worsens once serious complications develop.”*

In the Data Availability statement, “I” should be replaced with “We.” While restrictions on sharing human datasets are understandable, datasets can often be shared after removal of identifiers. The authors may need to reconsider this statement in light of publication transparency requirements.

Line 96-99: This sentence may need to be split. Also, remove the “-” before reference [1], which needs to be revised. The same for the preceding line.

The statement “As a biphasic infection” may imply there are other forms of the infection. Please revise to explicitly state that it is a biphasic disease, briefly explain the two phases, and continue to mention the effectiveness of antibiotic treatment in the acute stage.

110-111 ELISA and MAT are universally applied and not limited to low-resource settings. In any case, you would have more resources to maintain MAT than you would for PCR.

Line 129-132: Are you saying this outbreak marked the introduction of the PCR? If this is the case, I feel it fits better transferring it and merging it will line 114-118.

**Methods**

Line 135: I would re-write :”we conducted a cross-sectional study …” instead of “we adopted…

I suggest transferring line 150-156 to the section of data collection. You can then change the heading to “sample and data collection.

Line 150-153: I would re-write to clearly state these as reasons why 503 samples were collected and 492 tested, and here unsuccessful phlebotomy can`t be a reason since you mention the samples 503 were collected. Otherwise, explicitly mention in the results how many participants were enrolled, how many had their blood sampled, and how many of the samples were tested.

Line 172- put a reference to the data collection tool used so the reader can appreciate the questions used. This can even be the Excel file that you used to digitize the tool.

Line 175: redcap is symbolic for plain (tubes with no anticoagulant), so I recommend you replace redcap” accordingly.

Line 177: The protocol is incomplete if the incubation time at 37 is not specified. Also, quote the centrifuge speed in rcf, as rpm is not standard since it depends on the rotor radius. Or else, you may have to specify the centrifuge name, model, and manufacturer.

Line 183: more correct to say “…anti-Leptospira IgG and IgM **antibodies**

Line 202-212: the entire section needs to be revised. The first sentence could be rewritten without hyphens and with the actual Sigma reagents specified. This paragraph also contains methodologically incorrect statements that undermine the reliability of the MAT test results. For example, the authors mention maintaining the battery by subculturing in EMJH basal medium. It is not possible for Leptospira to grow in this basal medium unless it's been supplemented. There is no mention of the culture's age when it was used. The authors report an incubation time of 2-3 hours, suggesting significant variation in incubation times across samples. I also wonder why, for a seroepidemiological study like this, the authors test only up to a dilution of 1:160, yet titers above this can provide insights into the circumstances of exposure and could be used to detect probable cases of infection.

Statistical analysis

The authors performed 3 tests in parallel, yet in the risk factor analysis considered using the MAT, which undermines the rationale for conducting the two ELISAs in the first place. Additionally, the authors do not justify why the choice of the MAT results over the ELISAs

In line 225, it becomes incorrect to say “Descriptive statistics were used to describe sociodemographic characteristics and risk factors for leptospirosis”, since the MAT and, more so, the serum dilutions used can not be used to determine leptospirosis. It is rather limited to sero-exposure.

**RESULT**

The study analyses risk factors. However, some factors are not plausible for leptospirosis. Other reported risk factors lack contextual clarity. The 12-month recall period introduces substantial potential for recall bias and weakens interpretability. Some exposures are ambiguous; for example, drinking unboiled water: from what source?

Some figures repeat information already presented in the text and could be reduced. The relevance of geospatial maps should be reconsidered, as human movement means the site of sampling may not represent the site of exposure.

Lines 303–305 and 315–317 repeat content already described in the Methods.

334-336: The authors give seasonality as a reason for the contrast between their results and a previous study, which I think needs rethinking, since it is not possible that all the exposures happen within the September-October period when the authors conducted the study.

346: For the author's information, cross-reactions between Bratislava and Australis occur as these are both serovars in the same serogroup.

The conclusion should avoid referring to leptospirosis as disease occurrence, since this study measured serological exposure using single serum samples without concurrent clinical correlation. The conclusions should be framed in terms of **sero-exposure** rather than confirmed leptospirosis.
