# Supplementary material for: Seroepidemiology of human leptospirosis in subtropical Bhutan, 2024
Source: PLoS Negl Trop Dis. 2026 Nov 4;20(11):e0014821. doi: 10.1371/journal.pntd.0014821 (PMC13734453; doi:10.1371/journal.pntd.0014821)
Supplement: Supplementary file 4 [file pntd.0014821.s006.docx]

PNTD-D-26-00604
Sero-epidemiology of human leptospirosis in subtropical Bhutan, 2024
PLOS Neglected Tropical Diseases

Dear Dr. Shaden Kamhawi,

Thank you for taking time to review our manuscript. We addressed each review comment and they have definitely improved the quality of our manuscript and added more scientific vigor. Here, we would like to submit a point-to-point response letter to the reviewers and editors’ comments. The line numbers referred in this letter is of the clean version of our manuscript and may have changed from the original due to editions.

**Reviewer #1**

**Reviewer #1:** *This manuscript addresses an important and clearly under-studied topic: the sero-epidemiology of human leptospirosis in subtropical Bhutan. The topic is relevant to PLOS Neglected Tropical Diseases because leptospirosis is indeed neglected, diagnostically challenging, and highly sensitive to ecological and occupational conditions. The study also has practical public health value, since it attempts to quantify seroprevalence, describe circulating serovars, and identify behavioral and environmental risk factors in a setting where diagnostic capacity is limited. The sample size is respectable, the fieldwork appears substantial, and the effort to combine serology, spatial mapping, and risk-factor analysis is commendable. The headline finding, that roughly one in five participants had evidence of exposure by MAT, is potentially important for Bhutanese surveillance and clinical awareness.*

**Response:** Thank you for the positive note and pointing out the importance of our study.

**Reviewer #1:** *However, despite the manuscript’s relevance and the value of the dataset, I do not think the paper is yet suitable for acceptance in its current form. My overall recommendation is rejection in the present version, with encouragement to resubmit only after major methodological, analytical, and editorial reconstruction. The main problem is not that the study question is weak; rather, the current manuscript contains too many internal inconsistencies, imprecise interpretations, and presentation problems to support publication without substantial redevelopment.*

**Response**: Thank you for your recommendation. In this letter, we have the point-to-point responses to all reviewer comments. We thank the editors for the opportunity to revise our manuscript. All three reviewers’ comments and feedback were invaluable to revise the manuscript.

**Reviewer #1:** *The most serious concern is methodological clarity. The authors describe MAT as the reference test and use a cutoff titre of ≥1:80, justifying this on the basis that Bhutan is “not endemic” and that surveillance sensitivity should be enhanced. That rationale is not fully convincing, especially when the manuscript simultaneously argues that leptospirosis is an underrecognized but meaningful public health problem in the same setting. A lower threshold may increase apparent seroprevalence through reduced specificity, and the paper does not adequately discuss how this decision may inflate estimates or complicate comparison with other studies. Relatedly, the manuscript interprets MAT seropositivity as evidence that “one in five people were infected,” but in a cross-sectional serosurvey this wording is too strong. Seropositivity reflects serological evidence of exposure, not necessarily current or even recent infection. The paper repeatedly moves from “exposure” to “infection” without sufficient caution, and that overstatement affects both the author summary and the discussion.*

**Response:** We selected the cutoff titer of ≥1:80 because the objective of our study was seroprevalence estimation (evidence of previous exposure), not clinical confirmation of acute leptospirosis. For seroepidemiologic surveys, a cutoff of ≥1:80 is widely used to improve sensitivity for detecting prior exposure, especially in settings like Bhutan where baseline MAT titers are unknown. Higher thresholds such as ≥1:160 or ≥1:400 are more appropriate for diagnosing recent/acute infection because they increase specificity but reduce sensitivity for past exposure. Bhutan lacks established population-specific MAT reference titers and there are no nationally validated seroprevalence cutoffs; therefore, we adopted the commonly used ≥1:80 threshold for this seroprevalence study. This has been cautioned and described as a limitation of our study.

In addition, thank you for pointing out interchangeable use of “exposure” and “infection”. In the revised manuscript, we have been mindful and made changes throughout the manuscript to reflect that seropositivity as the evidence of past exposure, not necessarily current or even recent infection.

**Reviewer #1:** *The regression analysis also raises concerns. The manuscript states that variables with p < 0.200 in univariable analysis were entered into the multivariable model, but elsewhere the wording around the screening threshold is inconsistent, and the reporting of the final model is not fully coherent.*

**Response:** For this study, variables with p <0.200 in univariable analysis were entered into multivariable model. There was a typo in one place where it was typed p>0.200. We made the correction.

**Reviewer #1:** *In the narrative text, the authors say that primary education was “strongly associated” with seropositivity, yet the adjusted odds ratio given is 1.42 with p = 0.318, which is clearly not statistically significant. At the same time, tabled results show other retained variables such as rural residence, secondary education, and fever in the last 12 months, some of which are not emphasized consistently in the text. This gives the impression that the interpretation of the multivariable model was not handled carefully. In addition, the manuscript does not adequately discuss model diagnostics, collinearity, missingness handling, clustering by household or community, or the implications of substituting non-responders with nearest neighboring households. That substitution procedure may introduce selection bias, particularly in a community-based serosurvey.*

**Response:** Thank you for your comment. We acknowledge our mistake in stating primary education was “strongly associated” with seropositivity while OR = 1.42 with p = 0.318 and we adequately emphasized variables associated with seropositivity (**page 17, line 373 – 381**).

We performed additional statistical analysis. Variables with p < 0.200 in univariable logistic regression were considered candidates for multivariable analysis. To assess robustness of variable selection, five models were developed: (1) bidirectional stepwise selection, (2) full model, (3) backward elimination, (4) backward elimination with sex, settlement and education level forced into the model a priori as sociodemographic confounders, and (5) least absolute shrinkage and selection operator (LASSO) regression with variables informed by 10-fold cross-validation, followed by refitting of LASSO-selected variables as a standard unpenalized logistic regression. Model fit across resulting models was compared using the Akaike Information Criterion (AIC).

| **Model** | **N** | **AIC** |
| --- | --- | --- |
| Model 1: Stepwise both-direction (p<0.200) | 492 | 433.96 |
| Model 2: Full (p<0.200, no selection) | 492 | 437.79 |
| Model 3: Backward + Sociodemographic forced-in | 492 | 434.24 |
| Model 4: Backward (p<0.200) | 492 | 433.96 |
| Model 5: LASSO-selected (refit unpenalized) | 492 | 435.9 |

The model incorporating sex, settlement and level of education as forced sociodemographic covariates was adopted as the final model because these sociodemographic variables are established determinants of both exposure patterns and health-seeking behaviors in leptospirosis epidemiology, and their omission – even when not independently significant – risks residual confounding of the exposure-outcome associations of primary interest. Forcing these covariates into the model resulted in a negligible reduction in fit relative to the minimum-AIC alternative (= 0.28), indicating that robust confounder control could be achieved without meaningfully compromising model performance.

We also performed model diagnostics as reported in Methods (**page 12 – 13, line 291– 299**) and Results (**page 18, line 399– 415**).

Participants with incomplete or missing responses were re-contacted to obtain missing responses, resulting in a complete dataset for analysis (**page 9, line 208 – 209**)

From the selected participants, only 5 participants denied consent. Out of 5 participants, 3 were replaced by another member of the same household and 2 from neighbors. They constituted 1.0% (**page 8, line 195 – 202**). In addition, approaching the next *nearest* household still constituted randomized order

**Reviewer #1:** *There are also issues with sampling and representativeness. The study uses six subtropical communities in one district and relies partly on the 2017 census for one municipality because a current household registry was unavailable. That is understandable operationally, but it weakens the claim of precise sampling representativeness. The authors should be much more restrained in describing the sample as representative, even for the district, unless they can show how the sampling frame mismatch affected inclusion probabilities. Moreover, restricting participation to Bhutanese nationals because citizenship card numbers were used as identifiers may exclude potentially high-risk populations such as migrant or mobile workers. That is a meaningful epidemiologic limitation and should be acknowledged more explicitly because it could bias seroprevalence downward or alter the apparent occupational risk structure.*

**Response:** We used the data from 2017 census, which is the latest data available for Gelephu Thromde (municipality). This may not be as accurate as the household registry of the same year; this is the most reliable data available for sampling.

We acknowledge excluding non-Bhutanese as one of the limitations of this study and revised the manuscript accordingly (**page 23, line 497– 499**).

Regarding the sampling representativeness, 6 out of 12 blocks of the district were selected. The characteristics of 6 blocks included as shown below:

| **Study area** | **Altitude (masl)** | **Settlement** | **Border with India** |
| --- | --- | --- | --- |
| Chhudzom | 1169 – 1900 | Rural | No |
| Jigme Chhoeling | 372 – 1991 | Rural | No |
| Senggye | 249 – 900 | Rural | Yes |
| Shompangkha | 200 – 600 | Urban | Yes |
| Gelephu Thromde | 175 – 439 | Urban | Yes |
| Umling | 190 – 400 | Rural | Yes |

As such, two areas were urban areas; two were rural areas from lower altitude and two were rural areas from higher altitude. As the altitude of the district range from 175 to 3600 meters above the sea level (masl) with highest altitude of human settlement at 1900 – 2000 masl. The selected study areas may not be representative of the country but it represents the district.

**Reviewer #1:** *Another major weakness is the interpretation of serovars. The predominance of Australis and Lailike is interesting, but the authors themselves acknowledge that the antigen panel did not include Bratislava, which was highlighted in previous Bhutan data. That omission matters. With MAT, the apparent predominant serovar can be heavily shaped by the panel selected, and without a locally validated panel or molecular confirmation the serovar discussion remains suggestive rather than definitive.* *The statement that no cross-reactions occurred is also somewhat surprising in MAT-based work and deserves either additional explanation or a more cautious presentation. The discussion at several points moves beyond what the data can actually establish, especially when implying transmission pathways or reservoir ecology without concurrent animal or environmental sampling.*

**Response:** Thank you for this critical comment. Panel of live antigen used by the Royal Centers of Disease Control (RCDC) is WHO-recommended but not locally validated panel of serovars. As you pointed out that “serovar discussion remains suggestive rather than definitive”, we removed the particular paragraph from the Discussion. In other paragraphs also, we interpreted the findings cautiously so that the points do not move beyond what the data can actually establish.

For this study, cross-reactions were not reported because laboratory professionals at the Royal Centers for Disease Control reported only the primary reacting serovars. Therefore, we revised the term as “primary reacting serovars” (**page 15, line 326**).

**Reviewer #1:** *The manuscript also suffers from substantial writing and editing problems that go beyond routine copyediting. There are repeated grammatical errors, tense inconsistencies, article misuse, awkward phrasing, and occasional factual or logical slips. For example, the introduction contains a malformed sentence around the first documented evidence of human leptospirosis in Bhutan, and elsewhere the manuscript alternates between “infection,” “exposure,” and “seropositivity” as though they were interchangeable. There are also typographical and formatting issues in the tables, such as the erroneous “16 93.3)” entry for fishery farming, inconsistent spacing around percentages and p values, and several sentences that are syntactically incomplete or imprecise. These problems materially affect readability and scientific credibility. For a journal at this level, the manuscript would need a thorough language revision, not just minor polishing.*

**Response**: Thank you for your suggestions. We carefully revised the manuscript and we avoided interchangeable use of “infection”, “exposure” and “seropositivity”. As we reported the seroprevalence of leptospirosis, we used the term “seropositivity” throughout the manuscript.

We also corrected typographical errors and thoroughly revised the language and grammar for clarity and readability.

**Reviewer #1:** *I am also concerned by the data availability statement. The manuscript says that the original dataset is available from the corresponding author upon reasonable request, but then adds that national policy does not allow publication of the dataset on any platform. That may conflict with the journal’s data availability requirements, depending on whether a formal ethical or legal restriction can be documented and whether a controlled-access mechanism exists. As written, the statement appears weak for a PLOS journal and may become a publication barrier unless the authors provide a much more precise and policy-compliant explanation.*

**Response**: Thank you for pointing this out. We will make an anonymized dataset publicly available through two media. One, national repository which Bhutan will launch soon. Two, as a supplementary file (**S1 Dataset**) for this manuscript.

**Reviewer #1:** *That said, the study has genuine strengths and should not be dismissed as unimportant. The field data are valuable, the seroprevalence estimate is potentially consequential for Bhutan, and the identification of paddy farming, unboiled water consumption, and stagnant water contact as risk correlates is biologically plausible and regionally coherent. The paper could become publishable after extensive revision if the authors reframe their claims more conservatively, clean up the analysis, clarify the sampling design, improve the description and justification of MAT thresholds, correct inconsistencies between tables and text, and substantially revise the English. But that level of work is substantial enough that I would not classify this as a paper requiring only minor or moderate revision.*

**Response:** Thank you for your meaningful and constructive feedback. We revised the manuscript to clean up the analysis, clarify sampling design, improve description and justification of MAT threshold, made information in tables and text consistent. We also revised the grammar and tenses.

**Reviewer #1:** *My final decision is therefore to recommend rejection in the current form. This recommendation is based not on lack of relevance, but on the accumulation of methodological ambiguity, interpretive overreach, analytical inconsistency, and inadequate presentation. The underlying study appears potentially useful, but the manuscript as submitted does not yet meet the standard for acceptance.*

**Response:** Thank you for your recommendation. We thoroughly revised the manuscript trying to address all of your comments and feedback in addition to comments provided by two more reviewers. We anticipate that revised manuscript addresses your comments. Once again, thank you for your invaluable comments; they were helpful to revise our manuscript. In addition, we are willing to further revise the manuscript if there are additional comments in the future until our manuscript is suitable for publication in your journal.

**Reviewer # 2**

**Reviewer # 2:** *This manuscript addresses an important and understudied topic, and the seroprevalence estimates and contextual description of exposures are potentially informative for the region. However, there are substantial concerns that undermine confidence in the study's inferential components and require major revision before the work can be considered for publication.*

**Response:** Thank you for your kind words. We will try to address all of your comments. They were invaluable in revising the manuscript.

**Reviewer#2:** *Key issues relate to methodological clarity and analytical justification. The MAT procedures described raise questions about culture conditions, dilution ranges, and consistency of execution, which affect confidence in the reference test. The rationale for conducting ELISA alongside MAT is not carried into the analysis, as only MAT results are used for risk factor modeling without justification. Several exposure definitions lack operational clarity. Interpretation sometimes confuses serological exposure to clinical disease.*

**Response:** Thank you for this insightful comment. We have revised the manuscript to improve methodological clarity and analytical justification.

First, we expanded the description of the Microscopic Agglutination Test (MAT) procedures to provide additional details on culture conditions, serum dilution ranges, and quality assurance measures to ensure consistency of test execution. These revisions are presented in the Methods section (**pages 10 – 11, line 243 – 258**).

Second, we clarified the rationale for using both ELISA and MAT. ELISA was employed as a screening assay due to its practicality for large-scale serological surveys, while MAT was used as the reference test because of its higher specificity and ability to identify serovar-specific exposure. Since MAT is considered the reference standard for leptospirosis serology, risk factor analyses were modelled based on MAT-seropositivity. This rationale has now been explicitly stated in the Methods section (**Page 12, Line 274 – 279**).

Third, we revised the definitions of exposure variables to provide clear operational descriptions (**page 9, line 209 – 214**).

Finally, we revised the manuscript to ensure that serological evidence is consistently interpreted as indicating previous exposure to *Leptospira* spp. rather than clinical disease.

**Abstract and introduction**

**Reviewer#2:** *The background would benefit from clearer framing of the specific research gap being addressed in the context of the Bhutanese population. While the authors mention limited diagnostics as a constraint, it is not clearly explained why human sero-surveillance for leptospirosis in Bhutan is particularly important, nor how diagnostic limitations directly affect this investigation. Additionally, diagnostic test evaluation was not stated as an objective of the study, and no such evaluation appears in the analysis.*

**Response**: We revised the introduction section (**page 6, line 128 – 137**) to state the research gap. For diagnostic test evaluation, we intend to further analyze the data and write a separate paper.

**Reviewer#2:** *It should be clarified that MAT primarily determines* ***serogroups****, not definitive serovars, due to known cross-reactivities within serogroups.*

**Response**: Royal Centers for Disease Control (RCDC), the national reference laboratory, used the panel of 12 pathogenic serovars and one saprophytic serovar live antigen as presented in Table 1. As RCDC reported only the serovars with the highest titers, we used the term “primary reacting serovar”.

**Reviewer#2:** *The manuscript should also clarify whether there were positive participants for both IgM and IgG, how these were handled analytically, and how ELISA results compared with MAT findings.*

**Response**: For this paper, we considered MAT serostatus irrespective of ELISA because MAT is considered the gold standard reference test and provides higher specificity in identifying serogroup/serovar specific previous exposure.

**Reviewer#2:** The statement *“Leptospirosis can be treated with low-cost antibiotics if detected early but efficacy drops once serious complications like jaundice and organ failure develop”* would read more accurately as: *“…but prognosis worsens once serious complications develop.”*

**Response:** We revised the sentence.

**Reviewer#2:** *In the Data Availability statement, “I” should be replaced with “We.” While restrictions on sharing human datasets are understandable, datasets can often be shared after removal of identifiers. The authors may need to reconsider this statement in light of publication transparency requirements.*

**Response:** Thank you for pointing this out, we modified our statement. We will upload the anonymized dataset in the national repository once the platform is ready. The dataset will able to be made available publicly as supplementary material for this manuscript.

**Reviewer#2:** *Line 96-99: This sentence may need to be split. Also, remove the “-” before reference [1], which needs to be revised. The same for the preceding line.*

**Response**: We split both the sentences for clarity (**page 5, line 99 – 102**).

**Reviewer#2:** *The statement “As a biphasic infection” may imply there are other forms of the infection. Please revise to explicitly state that it is a biphasic disease, briefly explain the two phases, and continue to mention the effectiveness of antibiotic treatment in the acute stage.*

**Response**: We briefly described two phases of leptospirosis for clarity (**page 5, line 106 – 109**).

**Reviewer#2:** *110-111 ELISA and MAT are universally applied and not limited to low-resource settings. In any case, you would have more resources to maintain MAT than you would for PCR.*

**Response:** Thank you for your comment. We revised the sentence at **page 5, line 117 – 118.**

**Reviewer#2:** *Line 129-132: Are you saying this outbreak marked the introduction of the PCR? If this is the case, I feel it fits better transferring it and merging it will line 114-118.*

**Response:** We wanted to emphasize the importance of seroprevalence study of leptospirosis as the disease is increasingly becoming a public health threat. However, this outbreak did not directly result in the introduction of the PCR in the country.

**Methods**

**Reviewer#2:** Line 135: I would re-write: “we conducted a cross-sectional study …” instead of “we adopted…

**Response:** Thank you. We re-wrote the sentence as “we conducted a cross-sectional study” **(page 7, line 147).**

**Reviewer#2:** I suggest transferring line 150-156 to the section of data collection. You can then change the heading to “sample and data collection”.

**Response:** We moved the above-mentioned segment to “Sample and data collection” sub-section **(page 8, line 185).**

**Reviewer#2:** *Line 150-153: I would re-write to clearly state these as reasons why 503 samples were collected and 492 tested, and here unsuccessful phlebotomy can’t be a reason since you mention the samples 503 were collected. Otherwise, explicitly mention in the results how many participants were enrolled, how many had their blood sampled, and how many of the samples were tested.*

**Response:** We revised this sentence for clarity (**page 8, line 186 – 188**). Blood samples were collected from 503 participants. However, 11 samples couldn’t undergo laboratory test for one of the two reasons: (1) hemolyzed blood samples, and (2) inadequate sample volume.

**Reviewer#2:** *Line 172- put a reference to the data collection tool used so the reader can appreciate the questions used. This can even be the Excel file that you used to digitize the tool.*

**Response**: The questionnaire for this study was developed through brainstorming among authors and extensive literature review. We would like to share the questionnaire as **Supplementary file (S2 Table).**

**Reviewer#2:** *Line 175: redcap is symbolic for plain (tubes with no anticoagulant), so I recommend you replace redcap” accordingly.*

**Response:** Thank you and we used “plain tubes without anticoagulant” instead of “red cap” (**Page 9, Line 215 – 216**).

**Reviewer#2:** Line 177: *The protocol is incomplete if the incubation time at 37 is not specified. Also, quote the centrifuge speed in rcf, as rpm is not standard since it depends on the rotor radius. Or else, you may have to specify the centrifuge name, model, and manufacturer.*

**Response**: We reported incubation at 37-degree Celsius for 15 minutes in water bath for serum extraction and centrifugation at 1800 x g for 5 minutes (**page 9, line 217 – 219**). For your information, the centrifugation machine was manufactured by Clifton Range in South Korea (cat no: NE 030GTI).

**Reviewer#2:** *Line 183: more correct to say “…anti-Leptospira IgG and IgM* ***antibodies***

**Response:** We revised the sentence and added “antibodies” following the IgG and IgM (**Page 9, line 224).**

**Reviewer#2:** *Line 202-212: the entire section needs to be revised. The first sentence could be rewritten without hyphens and with the actual Sigma reagents specified. This paragraph also contains methodologically incorrect statements that undermine the reliability of the MAT test results. For example, the authors mention maintaining the battery by subculturing in EMJH basal medium. It is not possible for Leptospira to grow in this basal medium unless it's been supplemented. There is no mention of the culture's age when it was used. The authors report an incubation time of 2-3 hours, suggesting significant variation in incubation times across samples. I also wonder why, for a seroepidemiological study like this, the authors test only up to a dilution of 1:160, yet titers above this can provide insights into the circumstances of exposure and could be used to detect probable cases of infection.*

**Response**: Thank you for the critical feedback. We revised the sentence and added the information that live antigens were subcultured in **supplemented** EMJH base medium. The age of the culture at the time of serological testing for our study was 7 – 10 days. The incubation time was 3 hours for this study’s sample tastings. We incorporated all the changes in **page 10, line 243– 250.**

The test dilution was stopped at 1:160 because the primary objective of this study was to estimate seroprevalence and identify evidence of prior exposure to *Leptospira* spp. rather than to diagnose recent or acute infections. Consequently, MAT testing was performed up to a dilution of 1:160, which was considered sufficient for classifying seropositivity using the predefined cutoff titer of ≥1:80. We acknowledge that testing higher dilutions could have provided additional information on the distribution of antibody titers and may have helped identify individuals with stronger serological responses suggestive of more recent exposure or probable infection. However, budgetary constraints limited our ability to extend laboratory testing beyond the planned protocol.

**Statistical analysis**

**Reviewer#2:** *The authors performed 3 tests in parallel, yet in the risk factor analysis considered using the MAT, which undermines the rationale for conducting the two ELISAs in the first place. Additionally, the authors do not justify why the choice of the MAT results over the ELISAs*

**Response**: We performed three tests in parallel however MAT is considered for the risk factor analysis because MAT is the gold standard test for leptospirosis. As stated earlier, we intend to further analyze the data to determine the diagnostic accuracy of ELISA against the reference test MAT **(Page 12, Line 274 – 279).**

**Reviewer#2:** *In line 225, it becomes incorrect to say “Descriptive statistics were used to describe sociodemographic characteristics and risk factors for leptospirosis”, since the MAT and, more so, the serum dilutions used cannot be used to determine leptospirosis. It is rather limited to sero-exposure.*

**Response:** We appreciate your detailed observation. This sentence is revised the sentence. For example, “Descriptive statistics were used to describe sociodemographic characteristics and potential risk factors for seropositivity.” at **page 11-12, line 269 - 271.**

**RESULT**

**Reviewer#2:** *The study analyses risk factors. However, some factors are not plausible for leptospirosis. Other reported risk factors lack contextual clarity. The 12-month recall period introduces substantial potential for recall bias and weakens interpretability. Some exposures are ambiguous; for example, drinking unboiled water: from what source?*

**Response:** We thank the reviewer for this important observation. The study aimed to explore a broad range of demographic, environmental, and behavioral factors potentially associated with *Leptospira* seropositivity in a setting where epidemiological data are scarce. We agree that some variables may not represent direct biological risk factors for leptospiral exposure and have revised the manuscript to refer to them as "potential risk factors" for seropositivity rather than definitive risk factors of leptospirosis.

We also acknowledge that certain exposure variables, such as consumption of unboiled water, lacked detailed contextual information (e.g., source of water), which limited interpretation. We discussed this in **page 22, line 468 – 470.**

Furthermore, we recognize that the 12-month recall period may have introduced recall bias and exposure misclassification. This limitation has been added to the manuscript (**page 23, line 499 – 500),** and the findings are interpreted with appropriate caution. Future studies should collect more detailed exposure information and use shorter recall periods where feasible.

**Reviewer#2:** *Some figures repeat information already presented in the text and could be reduced. The relevance of geospatial maps should be reconsidered, as human movement means the site of sampling may not represent the site of exposure.*

**Response:** Thank you for this valuable suggestion. We have reviewed the figures and text and removed redundant information.

Regarding the geospatial maps, we agree that the location of sampling does not necessarily represent the location of exposure because participants may travel for work, education, or other activities. The maps were intended to illustrate the geographic distribution of sampled participants and seropositive individuals rather than to infer the exact locations of exposure.

**Reviewer#2:** *Lines 303–305 and 315–317 repeat content already described in the Methods.*

**Response:** Both lines 303-305 and 315-317 are deleted to avoid redundancy.

**Reviewer#2:** 334-336: *The authors give seasonality as a reason for the contrast between their results and a previous study, which I think needs rethinking, since it is not possible that all the exposures happen within the September-October period when the authors conducted the study.*

**Response**: The “seasons” referred to the season of symptoms/exposure, not the time of testing. In addition, the differences in seroprevalence between our study and previous reports may reflect variations in study populations, environmental exposures, geographic settings, and serological testing approaches **(page 20, line 426 – 429).**

**Reviewer#2:** 346: *For the author's information, cross-reactions between Bratislava and Australis occur as these are both serovars in the same serogroup.*

**Response:** Thank you for the information. We had to remove this paragraph during the revision. Also, we used “primary reacting serovars” to avoid ambiguity.

**Reviewer#2:** *The conclusion should avoid referring to leptospirosis as disease occurrence, since this study measured serological exposure using single serum samples without concurrent clinical correlation. The conclusions should be framed in terms of* ***sero-exposure*** *rather than confirmed leptospirosis.*

**Response**: We revised the Conclusion and framed in terms of sero-exposure rather than confirmed leptospirosis cases **(page 24 – 25, line 537 – 544).**

We take this opportunity to thank you for comprehensive review and comments. They were all meaningful and certainly helped us improve the quality of our manuscript.

**Reviewer#3**

**Reviewer #3:** *The manuscript is well organized and addresses an important public health issue in an understudied setting. The study design, laboratory methods, and statistical analyses are generally appropriate, and the findings contribute valuable baseline data on human leptospirosis in Bhutan. I recommend Minor Revision prior to acceptance, with the following editorial and data presentation improvements:*

**Response**: We thank you for your kind words.

**Reviewer #3:** *State an explicit study hypothesis in the Introduction. While the objectives are clearly described, a testable hypothesis would strengthen the scientific framework of the study.*

**Response**: We added hypothesis on **page 6, line 138 – 142.**

**Reviewer #3:** *Clarify the sample size calculation by providing the complete formula and assumptions used, including the expected prevalence, precision, design effect (if applicable), and justification for the anticipated dropout rate.*

**Response** We thank the reviewer for this comment. We have revised the Methods section to provide the complete sample size calculation, including the formula, expected seroprevalence (17.6%), desired precision (5%), confidence level (95%), study design effect (2.0), and justification for inflating the sample size by 5% to account for anticipated non-response and sample exclusions **(page 8, line 177 – 184).** We also included the formula for the sample size calculation (**page 7, line 170 – 175).** We have also clarified that the final sample was allocated across study sites using probability proportional to size sampling based on the household population.

**Reviewer #3:** *Correct inconsistencies in the description of variable selection for multivariable analysis. The Methods section states that variables with p < 0.20 were included in multivariable modeling, whereas the Results section states variables with p > 0.20 were included. This appears to be a typographical error and should be corrected.*

**Response:** There was typographical error. We corrected them and made consistent across the manuscript.

**Reviewer #3:** *Provide additional information regarding model diagnostics and goodness-of-fit for the final logistic regression model, if available.*

**Response**: Thank you; we included the extra information on model diagnostics in the Methods (**page 12 – 13, line 291– 299**) and Results (**page 18, line 399– 415**).

**Reviewer #3:** *Review Table 4 carefully. The statement that participants with primary education were “strongly associated” with seropositivity is not supported by the reported adjusted odds ratio (AOR 1.42; 95% CI 0.72–2.80; p = 0.318). The text should be revised to reflect statistical significance appropriately.*

**Response**: Thank you for pointing out this glaring error. We revised and removed this sentence.

**Reviewer #3:** *Improve figure readability, particularly the geospatial maps (Figure 5), by increasing font size, map labels, and resolution to ensure clarity in publication format.*

**Response:** We have submitted the Figure 5 with the resolution and file type as specified by the journal **(Fig 5)** using NAAS.

**Reviewer #3:** *Standardize formatting throughout the manuscript, including spacing around confidence intervals, p-values, percentages, and abbreviations.*

**Response:** Thank you; we adopted consistent format for CI, p-values, percentages, etc. throughout the manuscript.

**Reviewer #3:** *Review the manuscript for minor grammatical and typographical issues, including:
o “the first documented evidence of human leptospirosis outbreak a suspected outbreak”;
o “resulting in human” should be revised to “resulting in human infection” where appropriate;
o “expanding research capacity” in the Abstract should be revised to “expand research capacity” for parallel structure;
o several instances of missing articles, punctuation, and spacing should be corrected during copyediting.*

**Response**: We incorporated changes. For example, “In Bhutan, the first documented evidence of human leptospirosis was a suspected outbreak in the southwest district of Chukha in 2008.” **(page 5, line 112 – 113).** Likewise, we revised “resulting in human” to “resulting in human infection” where appropriate. We also revised “expanding research capacity” to “expand research capacity” **(page 4, line 77).** Throughout the manuscript, we revised the language, punctuation, articles and spaces.

**Reviewer #3:** *Strengthen the Data Availability Statement. The current statement indicates that data are available only upon reasonable request because of national policy restrictions. The authors should ensure that the statement fully complies with journal data-sharing requirements and clearly explains the legal or regulatory basis for these restrictions.*

**Response:** The anonymized data will be made available as a supplementary file (**S1 Dataset**). In addition, it will be uploaded in the national repository when platform is ready.

**Reviewer #3:** *Consider briefly discussing the potential impact of the MAT cutoff titer (1:80) on seroprevalence estimates and comparability with studies conducted in endemic settings using different thresholds.*

**Response**: We thank the reviewer for this suggestion. We have added a statement in the Discussion noting that the use of a MAT cutoff titer of ≥1:80, selected for seroepidemiological surveillance of exposure, may influence seroprevalence estimates and limit direct comparability with studies conducted in endemic settings that use higher diagnostic thresholds **(page 20, line 431 – 435).**

**Reviewer #3:** *Overall, the manuscript presents novel and policy-relevant findings, and the suggested revisions are intended primarily to improve clarity, transparency, and presentation rather than to address major scientific concerns.*

**Response:** Thank you for succinct comments. We tried to address them and they were invaluable to modify our manuscript.

Once again, we take this opportunity to thank the editors for the opportunity to revise the manuscript and reviewers for invaluable comments. We believe we adequately addressed each comment. However, we are willing to further revise the manuscript if there are any further comments from the reviewers.

Thanking you

Yours sincerely

**Monu Tamang**

On behalf of the co-authors
