# Supplementary material for: Seroepidemiology of human leptospirosis in subtropical Bhutan, 2024
Source: PLoS Negl Trop Dis. 2026 Nov 4;20(11):e0014821. doi: 10.1371/journal.pntd.0014821 (PMC13734453; doi:10.1371/journal.pntd.0014821)
Supplement: Supplementary file 5 [file pntd.0014821.s007.docx]

1 October 2026

PNTD-D-26-00604
Sero-epidemiology of human leptospirosis in subtropical Bhutan, 2024
*PLOS Neglected Tropical Diseases*

Dear Dr. Shaden Kamhawi,

Thank you for taking time to review our manuscript. We addressed each review comment, and they have improved the quality of our manuscript and added more scientific rigor. Here, we would like to submit a point-to-point response letter to the reviewer’s comments. The line numbers referred in this letter is of the clean version of our manuscript and may have changed from the original file due to editions.

**Reviewer#3**

**Reviewer#3**: *I thank the authors for bringing attention to this topic and addressing the reviewer concerns. I add the following comments for consideration:*

**Response:** Thank you for your kind words. We will address each of your comments.

**Reviewer#3**: *Line 110: Authors should state recommended/recognised antibiotic therapies. There is not much evidence for leptospirosis treatment using fluoroquinolones.*

**Response:** We removed fluoroquinolones from the list of recognized antibiotics therapies **(Page 6, Lines 132–134)**

**Reviewer#3**: *Line 225: The exact kit name for the ELISA kit from Gold Standard Diagnostics should be used here.*

**Response***:* The exact name of the ELISA test kit is *Leptospira* IgG ELISA **(Page 11, Line 251**)

**Reviewer#3**: *Lines 240, 244, 347 and references: Leptospira should be italicised as should all botanical names.*

**Response:** We italicized *Leptospira* (**Page 29, Line 624 & References**)

**Reviewer#3***: Line 257: Please add references as to why a cut-off of 1:80 was used.
Table 1 and in other places in the manuscript: Please change Uterecht to Utrecht, Grippotyphosa is in species L. kirschneri, Hardjoprajtno to Hardjoprajitno and Lailike to Lai-like*

**Response:** We added – **Rajapakse S, et al. "Seroprevalence of leptospirosis in an endemic mixed urban and semi-urban setting—A community-based study in the district of Colombo, Sri Lanka."** PLoS Neglected Tropical Diseases**. 2020;14(5):e0008309.** <https://doi.org/10.1371/journal.pntd.0008309> – for choosing cut-off of 1:80. (**Page 12, Lines 283–285; reference 16, Page 32, Lines 699–702)**

We revised the spelling mentioned above **(Table 1, Page 12; “Lai-like” also on Page 3, Line 63; Page 16, Line 372; Page 17, Line 386; Page 23, Line 475)**

**Reviewer#3**: *Statistical analysis: How was MAT serostatus defined for the logistic regressions...How was a seropositive (perhaps positive in at least one) and seronegative result (perhaps negative for all) determined considering several serovars were tested.*

**Response:** We added a brief definition of serostatus as “seropositive for at least one serovar among 13 serovars tested” **(Page 13, Lines 301–302)**.

**Reviewer#3**: *Lines 346 onwards: The comparisons made with other seroprevalence estimates should mention the diagnostic tests used and cut-offs.*

**Response:** We explicitly mentioned it as MAT-seropositivity (**Page 23, Lines 481–495**).

**Reviewer#3**: *Table 2: It seems to me “Fever in past 12 months”, “Participated in paddy plantation” and “Drink unboiled water”, "current symptoms", etc., are categorical variables, in which case presenting them as Yes/No with the numbers is better for the seroprevalence distribution tables. For something like “Fever in last 12 months” a Yes/No or Present/Absent is even necessary to interpret what 301 (61.2%) refers to.*

**Response:** All categorical variables were binary (Yes/No) therefore we reflected only “Yes” category where it is self-explanatory in such table. This decision was deliberate to keep the table succinct and avoid redundancy.

**Reviewer#3**: *Table 4: Same suggestion here. It would also help the reader if the significant categories were releveled/rebased to give positive (adjusted) odds ratios instead of these protective odds ratios for ease of interpretation. Also, please change “Referent” to “Reference” or “Ref” with a footnote all through the manuscript and supplementary information as it is standard.*

**Response**: Thank you for pointing this out. We set the reference categories for settlement (Rural) and education level (secondary) to the lowest-risk category to aid interpretation and used “reference” instead of referent in **Table 4 (page 22) and S1 Table** and Results (**page 19, lines 420–425; page 20, lines 442–448**) and Discussion (**page 24, lines 505–511 and 514–517**).

**Reviewer#3***: Line 468: Oral transmission of leptospirosis is likely ineffective due to protection from saliva, intact oral mucosa and gastric acid according to evidence from experimental studies on animal models. The discussion should therefore reflect this by showing possibility of past exposure through broken skin and interrupted mucous membranes and its relationship to fetching or drinking untreated water.*

**Response**: Thank you for the suggestion. We added this in the Discussion section (**page 25, lines 535–538**) and added a reference “Asoh T, Saito M, Villanueva SY, Kanemaru T, Gloriani N, Yoshida S. Natural defense by saliva and mucosa against oral infection by Leptospira. Can J Microbiol. 2014 Jun;60(6):383-9. doi: 10.1139/cjm-2014-0016. Epub 2014 Apr 24. PMID: 24861456.” (Reference 41; page 34, lines 772–774)

**Reviewer#3**: *Figure 3: please add confidence intervals to these estimates in the figure*

**Response:** We added confidence intervals in the **Figure 3.**

**Reviewer#3**: *Figure 4: It is hard to discern which slice belongs to which serovar in this pie chart. I suggest clearer presentation of the data here.*

**Response:** We used monochromatic color shade in this pie chart. As chart is built with color gradient and labels presented in sequential color gradient making it easier for readers to understand the chart. Thank you.

**Methods**

**Reviewer #3:** *1. Overview and Context. As the reviewer evaluating the Methods section of this manuscript on Leptospira seroepidemiology in subtropical communities (specifically in Sarpang), the overall assessment indicates that the manuscript is largely acceptable after revision. The authors have thoughtfully addressed most of the initial methodological concerns, particularly regarding the distinction between past serological evidence of exposure versus acute infection, framing appropriate inclusion/exclusion criteria, and securing the necessary ethical approvals.*

**Response:** Thank you for the kind comments. We appreciate your generosity.

**Reviewer #3:** *However, before final acceptance and publication, attention must be paid to a few remaining technical refinements, primarily centering around statistical handling of clustered sampling and the interpretation of sparse exposure data.
2. Detailed Breakdown by Criterion. Objectives & Testable Hypothesis:
Current Status: Yes. The objectives are clear (estimating seroprevalence, identifying circulating serovars, mapping cases, assessing risk factors). A testable hypothesis linking environmental/occupational contact to MAT seropositivity has been incorporated.
Recommendation: Maintain this explicit focus in the Introduction. Ensure that the primary hypothesis remains tightly coupled with the final multivariable regression modeling.*

**Response**: We thank the reviewer for this assessment. We retained Introduction's hypothesis statement (that environmental and occupational contact with potentially contaminated water or soil would be associated with seropositivity) unchanged. In addition, to tightly couple this hypothesis to the final multivariable model, we revised the opening paragraph of the Discussion to state explicitly that the observed associations for paddy plantation participation and drinking unboiled water provide empirical support for this a priori hypothesis (**Page 23, Lines 472–480**).

**Reviewer #3:** *Study Design: Current Status: Yes. The cross-sectional seroepidemiological design is appropriate for estimating prevalence and exploring associations, with correct explicit acknowledgment that temporality cannot be established. Recommendation: Continue to rigorously distinguish between seroprevalence/exposure and acute infection throughout the text.*

**Response:** Thank you and we ensure that exposure and acute infection are rigorously distinguished throughout the manuscript.

**Reviewer #3:** *Study Population & Representativeness: Current Status: Yes, with limitations. Adults ≥18 years residing in the study areas for ≥1 year were sampled using random household selection (one adult per household across six communities). Recommendation: Explicitly keep the noted limitation that excluding non-Bhutanese residents may impact generalizability, as migrant or foreign workers could possess distinctly different occupational exposure profiles.*

**Response:** Thank you, we revised the sentence to include “migrants, and foreign workers could possess distinctly different occupational exposure profiles” **(Page 26, Lines 564–566).**

**Reviewer #3:** *Sample Size Justification: Current Status: Yes for primary objectives; caution for subgroups. Calculations accounted for an expected prevalence of 17.6%, 5% precision, 95% confidence, a design effect of 2.0, and 5% inflation (target n=469; final analyzed n=492 with 95 MAT-positive outcomes). Recommendation: Explicitly note that while 492 participants with 95 positive outcomes offer a reliable basis for the primary prevalence and overall model, individual subgroup categories with very few events will inevitably generate unstable estimates.*

**Response**: We agree and have added this caveat directly to the Methods, where the achieved sample is first reported, rather than leaving it only implicit in the Results and Limitations (**Page 13, Lines 306–311).**

**Reviewer #3:** *Statistical Analyses: Current Status: Generally sound but requires strengthening. The use of conventional logistic regression, AIC-based model comparison, forced adjustment for core variables (sex, settlement, education), VIF/GVIF diagnostics, Hosmer–Lemeshow, ROC/AUC, and Cook’s distance sensitivity analyses are thorough. Recommendation: Address the clustered sampling structure. Because sampling occurred across six communities with household substitution, ordinary logistic regression risks underestimating standard errors due to intra-cluster correlation.*

**Response:** Thank you for the recommendation. We agree this was a gap in the original submission. We have added a new paragraph to the Statistical Analysis subsection that explicitly names the clustered design and explains how it was, and was not, accounted for at each stage of the study (**page 14, lines 321–330**).

As the paragraph now states, intra-cluster correlation was anticipated at the design stage through the design effect of 2.0 applied to the sample-size calculation, but the primary multivariable model itself used conventional maximum-likelihood standard errors.

**Reviewer #3:** *Analytical Consideration: Since there are only six communities, a random-effects mixed model may face convergence or cluster-count limitations. A cluster-robust standard error approach (at the community or sub-district level) is strongly recommended, alongside transparent descriptions of missing-data handling.*

**Response:** We agree. The Statistical Analysis subsection (**Page 14, lines 326–330)** states explicitly that neither standard cluster-adjustment approach was pursued, because six communities are too few to support either one reliably. A random-intercept mixed-effects logistic model risks non-convergence and unstable between-cluster variance estimates with so few upper-level clusters, and cluster-robust (sandwich-type) standard errors are similarly unreliable at this cluster count: the methodological literature generally recommends at least 20–50 clusters before cluster-robust inference can be trusted, even with small-sample corrections such as CR2 (Cameron & Miller, 2015; MacKinnon & Webb, 2017). We have therefore retained conventional maximum-likelihood standard errors for the primary model and now state this explicitly as a limitation, noting that a design effect of 2.0 was already incorporated at the sample-size stage to partially offset the anticipated loss of precision from clustering.

As mentioned in the “Sample and data collection” subsection, data completeness was reviewed after each field day, and participants were contacted within 24 hours to obtain missing information **(page 10, lines 231–232**).

**Reviewer #3:** *Ethical and Regulatory Compliance: Current Status: Yes. Approved by the Research Ethics Board of Health, Bhutan, and the Institutional Review Board of the International University of Health and Welfare, alongside institutional clearances and documented written/thumb-impression consent. Recommendation: Ensure final manuscript versions retain all clearance numbers and precise consent descriptions.*

**Response**: We have clearly mentioned about the ethics clearance and written consent in **page 15, lines 343–350**.

**Reviewer #3:** 3. Key Methodological Priorities Before Acceptance. Account for Clustered Sampling: Explicitly justify how the household/community sampling design was factored into the analysis. Implement cluster-robust standard errors if standard mixed-effects models are statistically infeasible due to the limited number of clusters (six communities).

**Response:** We agree this is the central methodological priority raised in the review. Regarding implementation: on closer methodological review, we do not believe cluster-robust standard errors can be justified either. With only six communities, cluster-robust (sandwich-type) variance estimators — even with the CR2 small-sample correction — fall short of the roughly 20–50 clusters generally considered necessary for reliable inference^[1],2^. We have therefore concluded that no standard cluster-adjustment method, whether a random-effects mixed model or cluster-robust standard errors, is statistically defensible at this cluster count.

Instead, we have retained the conventional maximum-likelihood standard errors used throughout the manuscript and have added an explicit statement to the Limitations noting that, with only six communities, intra-cluster correlation could not be adjusted for using any standard method, and that results should be interpreted with this constraint in mind (**Page 27, Lines 576–583**). We believe this transparent acknowledgement, together with the design effect already incorporated at the sample-size stage, appropriately addresses the reviewer’s concern given the constraints of the data.

**Reviewer #3:** *Cautious Handling of Sparse Data & Influential Observations: With 95 MAT-positive events against several predictors, certain categories showed extreme sensitivity (e.g., influential data point removal shifting paddy-farming odds ratios dramatically and making fever hospitalization inestimable). Do not add unnecessary extra predictors. Keep the final model parsimonious and emphasize cautious interpretation of sparse cells.*

**Response:** Thank you for your recommendation. We performed additional statistical analysis. Variables with p < 0.200 in univariable logistic regression were considered candidates for multivariable analysis. To assess robustness of variable selection, five models were developed: (1) bidirectional stepwise selection, (2) full model, (3) backward elimination, (4) backward elimination with sex, settlement and education level forced into the model a priori as sociodemographic confounders, and (5) least absolute shrinkage and selection operator (LASSO) regression with variables informed by 10-fold cross-validation, followed by refitting of LASSO-selected variables as a standard unpenalized logistic regression. Model fit across resulting models was compared using the Akaike Information Criterion (AIC).

| **Model** | **N** | **AIC** |
| --- | --- | --- |
| Model 1: Stepwise both-direction (p<0.200) | 492 | 433.96 |
| Model 2: Full (p<0.200, no selection) | 492 | 437.79 |
| Model 3: Backward + Sociodemographic forced-in | 492 | 434.24 |
| Model 4: Backward (p<0.200) | 492 | 433.96 |
| Model 5: LASSO-selected (refit unpenalized) | 492 | 435.9 |

The model incorporating sex, settlement and level of education as forced sociodemographic covariates was adopted as the final model because these sociodemographic variables are established determinants of both exposure patterns and health-seeking behaviors in leptospirosis epidemiology, and their omission – even when not independently significant – risks residual confounding of the exposure-outcome associations of primary interest. Forcing these covariates into the model resulted in a negligible reduction in fit relative to the minimum-AIC alternative (= 0.28), indicating that robust confounder control could be achieved without meaningfully compromising model performance.

**Reviewer #3:** *Exclusion of Diagnostic Validation (ELISA vs. MAT): Support the decision to exclude a comprehensive ELISA-vs-MAT diagnostic accuracy analysis from this manuscript, preserving the paper's focus strictly on seroepidemiology rather than assay validation.*

**Response**: Thank you for the suggestion. We revised the sentence to explicitly state that this paper is focused strictly on seroepidemiology rather than assay validation (**page 13, lines 304–305**).

**Results**

**Reviewer #3:** *Does the analysis presented match the analysis plan?
Yes. The results faithfully execute the analytical framework outlined in the methods. They progress logically from descriptive statistics and seroprevalence estimates to univariable screening, AIC-based multivariable model comparison, and rigorous diagnostic checks. The decision to focus strictly on MAT-confirmed seroprevalence (while systematically presenting ELISA comparisons) and defer diagnostic validation to a separate paper is consistently maintained.*

**Response:** Thank you so much.

**Reviewer #3:** *Are the results clearly and completely presented? Yes. Participant flow is fully transparent (503 enrolled, 11 excluded due to hemolysis/insufficient volume, leaving 492 analyzed). The data clearly show a MAT-confirmed seroprevalence of 19.3%, with Australis (49%) and Lailike (13%) as predominant serovars, and geographic clustering in Gelephu Thromde. Key independent risk predictors—paddy farming (aOR: 4.74) and drinking unboiled water (aOR: 2.34)—are clearly stated.*

**Response:** Thank you so much.

**Reviewer #3:** *Are the figures and tables of sufficient quality for clarity? Yes. The workflow diagram (Fig 2) cleanly traces sample processing and denominator accountability, and the geospatial map (Fig 1) effectively illustrates community distribution with proper copyright and licensing attributions. Tabular data are structured cleanly with exact confidence intervals and p-values.*

**Response:** Thank you so much.

**Reviewer #3:** *The Results section is rigorous, fully aligned with the study objectives and analysis plan, and presented with high clarity. No major modifications are required.*

**Response:** Thank you so much.

**Conclusions**

**Reviewer #3:** *1. Are the conclusions supported by the data presented? Assessment: Yes.
Details: The primary conclusion—that approximately one in five adults (19.3%) in subtropical Bhutan has serological evidence of prior Leptospira exposure—is directly derived from the MAT-confirmed prevalence data (n=95 out of 492). The identification of key risk drivers (paddy farming and drinking unboiled water) directly matches the significant associations found in the multivariable logistic regression models.
The assertion that human exposure is more common than previously recognized is well-supported given the historical backdrop of underdiagnosis and limited surveillance data in the region.*

**Response:** Thank you for the kind words.

**Reviewer #3:** 2. Are the limitations of analysis clearly described? Assessment: Yes.
Details: The authors appropriately highlight the cross-sectional design, explicitly acknowledging that temporality cannot be established between the identified risk factors and the seropositive outcomes. They transparently account for potential recall bias regarding self-reported exposures over the preceding 12 months. Although implicit in the study setup, the text could benefit from a brief final reinforcement of the sparse category constraints and community clustering limitations evaluated in the methods, but overall, the core epidemiological limitations are honestly stated.

**Response:** We thank the reviewer for this positive overall assessment and agree with the specific suggestion. We have added a sixth, concluding point to the Limitations paragraph that explicitly reinforces both constraints together (**page 27, lines 576–583**).

**Reviewer #3:** *3. Do the authors discuss how these data can be helpful to advance our understanding of the topic under study? Assessment: Yes. Details: The manuscript successfully bridges local empirical data with broader epidemiological understanding. By establishing baseline seroprevalence and mapping circulating serovars (predominantly Australis and Lailike with widespread distribution of Patoc), the study moves Bhutan past the bottleneck of sporadic outbreak-only reporting. It provides a foundational epidemiological profile for a high-risk subtropical environment, expanding our comprehension of zoonotic spillover risks in regions where leptospirosis clinically overlaps with other endemic tropical infections like dengue, malaria, and scrub typhus.*

**Response**: Thank you.

**Reviewer #3:** *4. Is public health relevance addressed? Assessment: Yes, exceptionally well. Details: The conclusion translates the data into four concrete, actionable public health recommendations: Educating communities on preventive behaviors.
Promoting the use of protective gear during high-risk agricultural activities (such as paddy farming).
Expanding research and surveillance capacity.
Strengthening hospital-based diagnostics to enable early antibiotic treatment and reduce severe complications.
These points directly target the structural gaps identified in the introduction and demonstrate high translational value for public health policy in Bhutan.*

**Response:** Thank you for calling public health relevance of this study as “exceptionally well.”

**Reviewer #3:** *Reviewer Conclusion on the Conclusions
The Conclusions section is robust, tightly anchored to the empirical findings, honest about study limitations, and highly impactful regarding public health translation. No major revisions are required for this section.*

**Response**: Thank you so much.

**Editorial and Data Presentation Modifications?**

Use this section for editorial suggestions as well as relatively minor modifications of existing data that would enhance clarity. If the only modifications needed are minor and/or editorial, you may wish to recommend “Minor Revision” or “Accept”.

**Reviewer #3:** *Clarify Sample Size Denominator Terminology in Text:
Ensure that the distinction between the total enrolled cohort (n=503), the excluded samples due to hemolysis/inadequate volume (n=11), and the final analyzed cohort (n=492) is consistently stated across the Abstract, Methods, and Results to prevent any reader confusion regarding denominator shifts.*

**Response:** Thank you for pointing this out. We include this information in the Results (**page 15, line 353**) to make it uniform with the Abstract and Methods.

**Reviewer #3:** *Refine Subgroup Data Description: In the risk factor and multivariable results text, briefly reinforce that while the primary model is robust, individual exposure categories with sparse positive counts (e.g., specific water or high-risk sub-exposures) should be interpreted with caution. This aligns with the sensitivity analyses performed regarding influential data points.*

**Response**: We thank the reviewer for this insightful suggestion. We have updated the text under the **Potential Risk Factors for MAT Seropositivity** section to clarify this point (**page 19, lines 433–437; page 21, lines 463–466**).

**Reviewer #3:** *Geospatial and Labeling Polish (Figure 1): Ensure that the boundaries and text annotations on the map (Figure 1) remain fully legible when formatted into the final multi-column journal layout, verifying that district names and legend items contrast cleanly against the Esri World Imagery basemap.*

**Response:** Thank you for your valuable comment. We confirm that labels are legible and image quality and specifications were assessed using the PLOS’ NAAS tool and met all the required specifications.

**Reviewer #3:** Minor Copyediting & Grammar Tweaks: Introduction/Methods: Check minor phrasing items, such as ensuring uniform verb tense when describing the execution of the 2024 field survey versus subsequent laboratory processing.

**Response:** We reviewed the Introduction and Methods for tense consistency between the narrative past tense used for study-specific procedures (the 2024 field survey and subsequent laboratory processing) and the present tense appropriately used for standing, general-fact statements (e.g., “ELISA and MAT are standard diagnostic methods”; manufacturer kit specifications).

In the Serological testing subsection, “The MAT … detected both IgM- and IgG-class antibodies” has been changed to the present tense “detects,” consistent with the general-capability statements used elsewhere, and distinct from the past-tense narrative describing what was specifically done in this study’s testing pathway. We also corrected a minor parallelism error in the same section (“9–11 indeterminates” to “9–11 indeterminate,” matching “positive”/“negative”).

**Reviewer #3:** *Conclusion/Public Health Recommendations: The four bulleted public health interventions are strong and actionable; ensure they mirror the precise wording used between the abstract summary and the final concluding paragraphs for maximum rhetorical impact. Overall Recommendation: The manuscript is in exceptional shape. Implementing these minor editorial polishes will ensure complete clarity for the readership of PLOS Neglected Tropical Diseases.*

**Response:** Thank you for your insightful comment. We revised the recommendations to mirror the precise wording used between the abstract summary and final concluding paragraphs **(Abstract: Page 4, Lines 76–81; Author Summary: Page 4, Line 95 to Page 5, Line 98; Conclusion: Page 28, Line 618 to Page 29, Line 623).**

**Summary and General Comments**

**Reviewer #3:** 1. *Overview and Evaluation Verdict. This manuscript presents a rigorous, timely, and epidemiologically sound investigation into the seroprevalence and associated risk factors of human leptospirosis in subtropical Bhutan. Utilizing a probability-proportional-to-size (PPS) sampling framework across six communities in the Sarpang district, the authors successfully bridge a critical knowledge gap in a region where leptospirosis is heavily under-recognized due to diagnostic constraints and clinical overlap with other endemic tropical infections (such as dengue, malaria, and scrub typhus). Overall, the manuscript exhibits high scholastic quality, transparent reporting, and exceptional public health relevance. The study is well-executed, the laboratory diagnostics (combining IgM/IgG ELISA screening with the gold-standard Microscopic Agglutination Test across a robust 13-serovar panel) are meticulously performed, and the statistical framework is sophisticated—incorporating multiple multivariable specifications, AIC model comparisons, collinearity diagnostics, and sensitivity analyses.*

**Response:** We sincerely thank the reviewer for their overwhelmingly positive assessment and thorough evaluation of our work. We are deeply grateful for the recognition of our study's methodological rigor, diagnostic methodology, and public health relevance to subtropical Bhutan. We appreciate these encouraging comments and have carefully addressed all remaining minor feedback in the manuscript to further enhance its clarity and impact.

**Reviewer #3:** *2. Novelty and Significance. Filling a Critical Evidence Gap: To the best of our knowledge, this is the first comprehensive human seroprevalence study conducted in this high-risk subtropical zone of Bhutan. Historically, national surveillance has relied on passive reporting or outbreak-driven tracking (such as the significant 2025 outbreak). Establishing a baseline seroprevalence of 19.3% (approximately one in five adults) fundamentally reshapes our understanding of disease endemicity, demonstrating that asymptomatic or mild, unrecognized exposure is far more common than previously appreciated.*

**Response**: We express our appreciation to the reviewer for highlighting the novel contribution and public health significance of our study. We agree that establishing this baseline seroprevalence (19.3%) is vital for transitioning beyond passive and outbreak-driven reporting toward a proactive, evidence-based approach to leptospirosis management in Bhutan.

**Reviewer #3:** *Actionable Epidemiological Mapping: The identification of predominant serovars (Australis at 49% and Lailike at 13%) alongside geospatial clustering within Gelephu Thromde provides local health authorities with invaluable targets for vector/reservoir control and resource allocation.*

**Response:** Thank you very much.

**Reviewer #3:** *Translational Public Health Value: The study avoids purely academic abstraction by directly linking its empirical findings—specifically paddy farming and drinking unboiled water as independent risk drivers—to four concrete, highly actionable public health recommendations (community education, protective occupational gear, diagnostic expansion, and regional surveillance strengthening).*

**Response**: Thank you so much for finding translational public health value in our recommendations.

**Reviewer #3:** 3. *Major Strengths of the Study. Rigorous Sampling Design: The implementation of PPS sampling based on district registries, paired with an a priori sample size calculation accounting for a design effect (2.0) and non-response inflation, ensures strong internal validity and representativeness of the target population.*

**Response:** Thank you for finding rigorous sampling design as the major strength of our study.

**Reviewer #3:** *Methodological Transparency: The analytical approach is exemplary. By testing multiple model selection strategies (bidirectional, backward, forced sociodemographic control, and LASSO cross-validation) and rigorously reporting diagnostic checks (VIF/GVIF, Hosmer-Lemeshow, ROC/AUC, and Cook's distance), the authors demonstrate exceptional analytical rigor.*

**Response**: Thank you very much for finding our analytical approach as “exemplary” and “exceptional analytical rigor.”

**Reviewer #3:** *Pragmatic Diagnostic Scope: The authors made the correct methodological decision to focus primarily on MAT-confirmed seroprevalence for risk factor modeling while systematically presenting ELISA comparisons, and properly deferring a full ELISA-vs-MAT diagnostic validation to a separate dedicated manuscript.*

**Response**: Thank you very much. We focused strictly on seroepidemiology in this paper and kept diagnostic accuracy for another manuscript.

**Reviewer #3:** *4. Limitations and Areas for Careful Interpretation. As transparently acknowledged by the authors: Cross-Sectional Constraints: The cross-sectional design inherently precludes establishing temporality between self-reported exposures (over the preceding 12 months) and MAT seropositivity. Recall Bias & Sparse Categories: Self-reported behavioral exposures are susceptible to recall bias. Furthermore, certain sub-exposures suffered from sparse positive event counts, meaning that multivariable effect sizes (such as those observed in sensitivity checks upon removing influential observations) must be interpreted with appropriate conservatism.*

**Response**: We revised the manuscript and included a sentence on need of cautious interpretation when it comes to sparse positive event counts (**page 27, lines 570–576)**.

**Reviewer #3*:*** *Clustered Sampling Considerations: While ordinary logistic regression was thoroughly evaluated, the authors should ensure that standard error estimations appropriately account for intra-cluster correlation across the six sampled communities or clearly state this as a minor analytical boundary.*

**Response**: As detailed in our responses to previous comments, we have explicitly justified, in the Methods, why conventional standard errors were retained for the primary model: with only six communities, neither a random-effects mixed model nor cluster-robust (sandwich-type) standard errors can be reliably estimated, so no standard cluster-adjustment method is statistically defensible. The corresponding analytical boundary is now clearly stated as the sixth point of the Limitations (**page 27, lines 576–583**). We believe this transparently addresses the reviewer’s request to either account for clustering directly or clearly flag it as a boundary of the analysis.

**Reviewer #3:** *5. Ethics, Regulatory, and Publication Compliance
Research Ethics: The study demonstrates full compliance with ethical and regulatory requirements, holding formal approvals from the Research Ethics Board of Health (REBH), Bhutan, and the Institutional Review Board of the International University of Health and Welfare (IUHW), Japan, alongside administrative site clearances.
Informed Consent: Ethical acquisition of written consent (including witnessed thumb impressions for non-literate participants) is fully documented.*

**Response:** Thank you for acknowledging that all ethics clearance were secured and well documented in the manuscript.

**Reviewer #3:** *Data Availability: The manuscript complies with open science standards, providing clear statements regarding underlying data access through institutional contact points and upcoming national repositories.*

**Response:** The dataset will be kept open as supplementary file. Meanwhile, permission to use the dataset can be availed from Health and Medical Research Centre (HMRC) at CRRH. We also plan to upload the file in the national repository when it is launched.

**Reviewer #3:** *6. Final Recommendation. The manuscript is exceptionally well-prepared and meets all necessary criteria for publication in PLOS Neglected Tropical Diseases. No major new experiments or data collection are required. Final Verdict: Accept (or Minor Revision solely to accommodate the final minor editorial polishes regarding sample denominator clarity and cluster-robust standard error text documentation).*

**Response**: Thank you so much for extremely positive response. We look forward to having the manuscript published by the prestigious PLOS Neglected Tropical Diseases.

**Figure resubmission:**

*While revising your submission, we strongly recommend that you use PLOS’s NAAS tool (*[*https://ngplosjournals.pagemajik.ai/artanalysis*](https://ngplosjournals.pagemajik.ai/artanalysis)*) to test your figure files. NAAS can convert your figure files to the TIFF file type and meet basic requirements (such as print size, resolution), or provide you with a report on issues that do not meet our requirements and that NAAS cannot fix. After uploading your figures to PLOS’s NAAS tool -* [*https://ngplosjournals.pagemajik.ai/artanalysis*](https://ngplosjournals.pagemajik.ai/artanalysis)*, NAAS will process the files provided and display the results in the "Uploaded Files" section of the page as the processing is complete. If the uploaded figures meet our requirements (or NAAS is able to fix the files to meet our requirements), the figure will be marked as "fixed" above. If NAAS is unable to fix the files, a red "failed" label will appear above. When NAAS has confirmed that the figure files meet our requirements, please download the file via the download option, and include these NAAS processed figure files when submitting your revised manuscript.*

**Response:** Thank you for the suggestion. All the Figures that we submitted following the first round of revision were screened using PLOS’s NAAS tool and met the basic requirements. All the figures that we are re-submitting in this round of revision are also screened using NAAS and meet all the specified requirements.

**Reproducibility:**

 To enhance the reproducibility of your results, we recommend that authors of applicable studies deposit laboratory protocols in [protocols.io](http://protocols.io/), where a protocol can be assigned its own identifier (DOI) such that it can be cited independently in the future. Additionally, PLOS ONE offers an option to publish peer-reviewed clinical study protocols. Read more information on sharing protocols at <https://plos.org/protocols?utm_medium=editorial-email&utm_source=authorletters&utm_campaign=protocols>

**Response:** Thank you for the suggestion. We will deposit the laboratory protocols in protocols.io.

We take this opportunity to thank the editors and reviewers for the opportunity to revise the manuscript. We believe we adequately addressed each comment. However, we are willing to further revise the manuscript if there are any further comments from the reviewers.

Thanking you

Yours sincerely

**Monu Tamang**

On behalf of the co-authors

1. Cameron AC, Miller DL. A Practitioner's Guide to Cluster-Robust Inference. J Hum Resour. 2015;50(2):317–372.

   ^2^ MacKinnon JG, Webb MD. Wild bootstrap inference for wildly different cluster sizes. J Appl Econom. 2017;32(2):233–254.
