# Supplementary material for: Seroepidemiology of human leptospirosis in subtropical Bhutan, 2024
Source: PLoS Negl Trop Dis. 2026 Nov 4;20(11):e0014821. doi: 10.1371/journal.pntd.0014821 (PMC13734453; doi:10.1371/journal.pntd.0014821)
Supplement: Peer review history

# Peer review: Decision Letter 0

**DOI:** 10.1371/journal.pntd.0014821.r001

**Authors:** Ana LTO Nascimento, Martin Wainaina

11 Jun 2026

PNTD-D-26-00604

Sero-epidemiology of human leptospirosis in subtropical Bhutan, 2024

PLOS Neglected Tropical Diseases

Dear Dr. Tamang,

Thank you for submitting your manuscript to PLOS Neglected Tropical Diseases. After careful consideration, we feel that it has merit but does not fully meet PLOS Neglected Tropical Diseases's publication criteria as it currently stands. Therefore, we invite you to submit a revised version of the manuscript that addresses the points raised during the review process.

Please submit your revised manuscript within by Aug 10 2026 11:59PM. If you will need more time than this to complete your revisions, please reply to this message or contact the journal office at plosntds@plos.org. When you're ready to submit your revision, log on to https://www.editorialmanager.com/pntd/ and select the 'Submissions Needing Revision' folder to locate your manuscript file.

Please include the following items when submitting your revised manuscript:

* A letter that responds to each point raised by the editor and reviewer(s). You should upload this letter as a separate file labeled 'Response to Reviewers'. This file does not need to include responses to any formatting updates and technical items listed in the 'Journal Requirements' section below.

* A marked-up copy of your manuscript that highlights changes made to the original version. You should upload this as a separate file labeled 'Revised Manuscript with Track Changes'.

* An unmarked version of your revised paper without tracked changes. You should upload this as a separate file labeled 'Manuscript'.

If you would like to make changes to your financial disclosure, competing interests statement, or data availability statement, please make these updates within the submission form at the time of resubmission. Guidelines for resubmitting your figure files are available below the reviewer comments at the end of this letter.

As the corresponding author, your ORCID iD is verified in the submission system and will appear in the published article. PLOS supports the use of ORCID, and we encourage all coauthors to register for an ORCID iD and use it as well. Please encourage your coauthors to verify their ORCID iD within the submission system before final acceptance, as unverified ORCID iDs will not appear in the published article. Only the individual author can complete the verification step; PLOS staff cannot verify ORCID iDs on behalf of authors.

We look forward to receiving your revised manuscript.

Kind regards,

Martin Wainaina

Academic Editor

PLOS Neglected Tropical Diseases

Ana LTO Nascimento

Section Editor

PLOS Neglected Tropical Diseases

Shaden Kamhawi

co-Editor-in-Chief

PLOS Neglected Tropical Diseases

orcid.org/0000-0003-4304-636XX

Paul Brindley

co-Editor-in-Chief

PLOS Neglected Tropical Diseases

orcid.org/0000-0003-1765-0002

**Additional Editor Comments (if provided):**

**Journal Requirements:**

If the reviewer comments include a recommendation to cite specific previously published works, please review and evaluate these publications to determine whether they are relevant and should be cited. There is no requirement to cite these works unless the editor has indicated otherwise.

1) Please ensure that the CRediT author contributions listed for every co-author are completed accurately and in full.

At this stage, the following Authors/Authors require contributions: Monu Tamang, Rie Takeuchi, Thinley Dorji, Kinley Choki, Tshokey Tshokey, Chenga Dawa, Choki Dolkar, Kesang Wangchuk, Dorji Tshering, Sangay Tenzin, Kinley Wangchuk, Rinzin Wangchuk, Naoko Yamamoto, and Yasuyuki Kato. Please ensure that the full contributions of each author are acknowledged in the "Add/Edit/Remove Authors" section of our submission form.

The list of CRediT author contributions may be found here: https://journals.plos.org/plosntds/s/authorship#loc-author-contributions

2) We do not publish any copyright or trademark symbols that usually accompany proprietary names, eg ©,  ®, or TM  (e.g. next to drug or reagent names). Therefore please remove all instances of trademark/copyright symbols throughout the text, including:

- ® on pages: 13, and 14.

3) Please upload all main figures as separate Figure files in .tif or .eps format. For more information about how to convert and format your figure files please see our guidelines:

https://journals.plos.org/plosntds/s/figures

4) In the online submission form, you indicated that Original dataset is available from corresponding author upon reasonable request.  I have shared the original dataset during the manuscript submission purely for peer-review process. Policies in my country (Bhutan) does not allow publication of dataset in any platform.. All PLOS journals now require all data underlying the findings described in their manuscript to be freely available to other researchers, either

1. In a public repository

2. Within the manuscript itself

3. Uploaded as supplementary information.

This policy applies to all data except where public deposition would breach compliance with the protocol approved by your research ethics board. If your data cannot be made publicly available for ethical or legal reasons (e.g., public availability would compromise patient privacy), please explain your reasons by return email and your exemption request will be escalated to the editor for approval. Your exemption request will be handled independently and will not hold up the peer review process, but will need to be resolved should your manuscript be accepted for publication. One of the Editorial team will then be in touch if there are any issues.

5) We have amended your Competing Interest statement to comply with journal style. We kindly ask that you double check the statement and let us know if anything is incorrect.

**Reviewers' Comments:**

Reviewer's Responses to Questions

**Key Review Criteria Required for Acceptance?**

As you describe the new analyses required for acceptance, please consider the following:

**Methods**

-Are the objectives of the study clearly articulated with a clear testable hypothesis stated?

-Is the study design appropriate to address the stated objectives?

-Is the population clearly described and appropriate for the hypothesis being tested?

-Is the sample size sufficient to ensure adequate power to address the hypothesis being tested?

-Were correct statistical analysis used to support conclusions?

-Are there concerns about ethical or regulatory requirements being met?

Reviewer #1: (No Response)

Reviewer #2: The study’s objectives to estimate leptospiral sero-exposure and explore associated factors are generally stated, but explicit, testable hypotheses are not clearly articulated, and the risk factor component reads as exploratory rather than hypothesis-driven. The cross-sectional design is appropriate. The sample size appears adequate for prevalence estimation. There are some concerns with the statistical approach, including a lack of justification for selecting MAT alone for modeling despite multiple assays and unclear exposure definitions. While the descriptive findings are informative, the conclusions about determinants of exposure are not fully supported by the analyses as presented. Ethical approvals and consent procedures are well addressed.

Reviewer #3: -Yes. The objectives are clearly stated in the Introduction: (1) estimate human leptospirosis seroprevalence, (2) identify circulating serovars, (3) map seropositive cases, and (4) assess risk factors for leptospirosis. While an explicit hypothesis statement was not originally included, the study was designed under the hypothesis that leptospirosis exposure is present in subtropical Bhutan and is associated with specific environmental, behavioral, and occupational risk factors. To address this comment, we will revise the Introduction to explicitly state the study hypothesis.

-Yes. A community-based cross-sectional study design was appropriate for estimating seroprevalence, characterizing circulating serovars, describing geographic distribution, and identifying factors associated with seropositivity at the population level. The design aligns with the primary objectives of assessing the burden and epidemiological characteristics of leptospirosis in a previously understudied region.

-Yes. The study population was clearly defined as adults (≥18 years) residing for at least one year in six subtropical communities of Sarpang District, Bhutan. Inclusion and exclusion criteria were specified, and households were selected using probability-based sampling methods. This population is appropriate because it resides in an area with environmental conditions favorable for leptospirosis transmission and therefore provides a suitable population for evaluating exposure and associated risk factors.

-Yes. Sample size calculations were performed using an expected seropositivity of 17.6%, a 95% confidence level, and allowance for participant attrition. The required sample size was 466 participants, while 492 participants with complete laboratory results were included in the final analysis. Therefore, the achieved sample size exceeded the minimum calculated requirement and was considered adequate to estimate seroprevalence and evaluate major risk factors.

-Yes. Descriptive statistics were used to summarize participant characteristics. Logistic regression analyses were conducted to evaluate associations between risk factors and MAT-confirmed seropositivity. Variables meeting the predefined screening criterion (p < 0.20 in univariable analysis) were entered into multivariable logistic regression models. Model selection was guided by Akaike Information Criterion (AIC), and adjusted odds ratios with 95% confidence intervals were reported. These approaches are appropriate for identifying independent predictors of a binary outcome in a cross-sectional study. To further strengthen the manuscript, we will clarify model-building procedures and provide additional details regarding variable selection and model diagnostics in the revised Methods section.

-No. Ethical and regulatory requirements were appropriately addressed. The study received approval from the Research Ethics Board of Health, Bhutan (PO/RL/2024.13.MC) and the Institutional Review Board of the International University of Health and Welfare, Japan (23-Ac-1). Administrative clearances were obtained from relevant national and local authorities. Written informed consent was obtained from all participants, with alternative procedures (thumb impression and witness signature) used for participants unable to read or write. Therefore, the study complied with applicable ethical and regulatory standards.

**********

**Results**

-Does the analysis presented match the analysis plan?

-Are the results clearly and completely presented?

-Are the figures (Tables, Images) of sufficient quality for clarity?

Reviewer #1: (No Response)

Reviewer #2: The results are generally presented in a structured manner, but some findings repeat information already stated in the methods, and certain exposure variables lack sufficient clarity to allow full interpretation. Tables are readable but at times dense, and some figures (particularly maps) add limited interpretive value relative to the text.

Reviewer #3: -Yes. The analyses presented are consistent with the statistical analysis plan described in the Methods section. The study first used descriptive statistics to summarize participant characteristics, seroprevalence estimates, and exposure variables. Subsequently, univariable logistic regression was conducted to identify factors associated with MAT seropositivity, followed by multivariable logistic regression including variables meeting the predefined inclusion criterion (p < 0.20). Geographic mapping of seropositive cases was also performed as specified in the objectives. Overall, the reported analyses align with the stated analytical approach.

A minor clarification may be warranted regarding the model selection process and the rationale for retaining specific variables in the final multivariable model.

-Generally, yes. The results are organized logically and presented under clear subsections covering:

• Participant characteristics;

• Seroprevalence estimates using ELISA IgM, ELISA IgG, and MAT;

• Distribution of circulating serovars;

• Geospatial distribution of seropositive cases;

• Exposure to potential risk factors; and

• Univariable and multivariable regression analyses.

Key findings are reported with appropriate measures of effect, including prevalence estimates, odds ratios, 95% confidence intervals, and p-values. The results directly address each of the study objectives.

To further improve clarity, the authors may consider:

• Reporting the number of participants included in the final multivariable model;

• Providing additional information on model performance and goodness-of-fit;

• Clarifying the statement regarding variable selection for multivariable analysis (the manuscript states variables with p-value "> 0.2" were included, whereas the Methods section specifies p < 0.20, suggesting a typographical error).

-Yes, overall the tables and figures adequately support the presentation of results.

Strengths:

• Table 2 clearly summarizes participant characteristics.

• Table 3 effectively presents exposure variables and potential risk factors.

• Table 4 appropriately reports crude and adjusted odds ratios with confidence intervals and p-values.

• Figures 3 and 4 clearly illustrate seroprevalence estimates and serovar distribution.

• Figure 5 provides useful visualization of the geographic distribution of seropositive cases.

Areas for improvement:

• Increase the resolution and font size of geographic maps (Figure 5 panels) to improve readability in print format.

• Ensure consistent labeling and formatting across all figures and tables.

• Figure 2 (laboratory testing pathway) could be simplified graphically to improve readability.

• Consider adding sample sizes directly within figure legends where appropriate.

Overall, the figures and tables are of sufficient quality to support interpretation of the findings, with only minor formatting improvements recommended for publication.

**********

**Conclusions**

-Are the conclusions supported by the data presented?

-Are the limitations of analysis clearly described?

-Do the authors discuss how these data can be helpful to advance our understanding of the topic under study?

-Is public health relevance addressed?

Reviewer #1: (No Response)

Reviewer #2: The conclusions are only partially supported by the data. Seroprevalence findings are well documented, but some statements extend to implications about disease occurrence and risk factors that are not fully supported by the serological and cross-sectional data presented. The limitations of the analysis are mentioned but not sufficiently explored, particularly regarding the interpretation of seropositivity, exposure recall, and the constraints of the analytical approach. The authors place the findings within the broader context of leptospirosis epidemiology and highlight how the data contribute to understanding exposure in a previously understudied setting. Public health relevance is appropriately addressed, although the discussion would be strengthened by aligning closely with the scope of the findings.

Reviewer #3: -Yes. The conclusions are generally well supported by the study findings. The reported seroprevalence estimates demonstrate substantial exposure to Leptospira spp. in the study population, while the multivariable analysis identifies paddy farming, drinking unboiled water, and contact with stagnant water as significant risk factors. The conclusions appropriately emphasize the presence of leptospirosis in subtropical Bhutan, the diversity of circulating serovars, and the need for strengthened surveillance and diagnostic capacity. The authors avoid making causal inferences beyond what can be supported by a cross-sectional study design.

-Yes. The manuscript provides a balanced discussion of several important limitations, including:

• The exclusion of animal populations from serological testing, limiting assessment of transmission pathways;

• Limitations of MAT, including delayed seroconversion and variability in interpretation;

• Potential underestimation of seroprevalence due to the limited serovar panel;

• The absence of PCR-based diagnostics, which may have identified acute infections; and

• Limited generalizability of findings beyond the study district.

These limitations are appropriately acknowledged and discussed in relation to the interpretation of the results.

-Yes. The authors clearly explain how the findings contribute to the understanding of leptospirosis epidemiology in Bhutan. The study provides updated seroprevalence estimates, identifies predominant circulating serovars, describes geographic distribution patterns, and identifies key environmental and occupational risk factors. As one of the few human sero-epidemiological studies conducted in Bhutan, the findings establish an important baseline for future surveillance, molecular investigations, and One Health research initiatives.

-Yes. Public health relevance is a major strength of the manuscript. The authors appropriately discuss the implications of their findings for:

• Strengthening diagnostic capacity at regional hospitals;

• Improving surveillance systems for leptospirosis;

• Promoting early diagnosis and treatment to reduce severe disease and mortality;

• Implementing targeted health education and preventive interventions among high-risk populations, particularly paddy farmers;

• Encouraging protective behaviors such as the use of boots, gloves, and safe drinking water practices; and

• Enhancing preparedness in the context of climate change and increasing risk of water-borne zoonotic diseases.

The discussion effectively links the study findings to actionable public health recommendations and demonstrates their relevance for disease prevention and control in Bhutan and similar endemic settings.

**********

**Editorial and Data Presentation Modifications?**

Use this section for editorial suggestions as well as relatively minor modifications of existing data that would enhance clarity. If the only modifications needed are minor and/or editorial, you may wish to recommend “Minor Revision” or “Accept”.

Reviewer #1: (No Response)

Reviewer #2: (No Response)

Reviewer #3: The manuscript is well organized and addresses an important public health issue in an understudied setting. The study design, laboratory methods, and statistical analyses are generally appropriate, and the findings contribute valuable baseline data on human leptospirosis in Bhutan. I recommend Minor Revision prior to acceptance, with the following editorial and data presentation improvements:

1. State an explicit study hypothesis in the Introduction. While the objectives are clearly described, a testable hypothesis would strengthen the scientific framework of the study.

2. Clarify the sample size calculation by providing the complete formula and assumptions used, including the expected prevalence, precision, design effect (if applicable), and justification for the anticipated dropout rate.

3. Correct inconsistencies in the description of variable selection for multivariable analysis. The Methods section states that variables with p < 0.20 were included in multivariable modeling, whereas the Results section states variables with p > 0.20 were included. This appears to be a typographical error and should be corrected.

4. Provide additional information regarding model diagnostics and goodness-of-fit for the final logistic regression model, if available.

5. Review Table 4 carefully. The statement that participants with primary education were “strongly associated” with seropositivity is not supported by the reported adjusted odds ratio (AOR 1.42; 95% CI 0.72–2.80; p = 0.318). The text should be revised to reflect statistical significance appropriately.

6. Improve figure readability, particularly the geospatial maps (Figure 5), by increasing font size, map labels, and resolution to ensure clarity in publication format.

7. Standardize formatting throughout the manuscript, including spacing around confidence intervals, p-values, percentages, and abbreviations.

8. Review the manuscript for minor grammatical and typographical issues, including:

o “the first documented evidence of human leptospirosis outbreak a suspected outbreak”;

o “resulting in human” should be revised to “resulting in human infection” where appropriate;

o “expanding research capacity” in the Abstract should be revised to “expand research capacity” for parallel structure;

o several instances of missing articles, punctuation, and spacing should be corrected during copyediting.

9. Strengthen the Data Availability Statement. The current statement indicates that data are available only upon reasonable request because of national policy restrictions. The authors should ensure that the statement fully complies with journal data-sharing requirements and clearly explains the legal or regulatory basis for these restrictions.

10. Consider briefly discussing the potential impact of the MAT cutoff titre (1:80) on seroprevalence estimates and comparability with studies conducted in endemic settings using different thresholds.

Overall, the manuscript presents novel and policy-relevant findings, and the suggested revisions are intended primarily to improve clarity, transparency, and presentation rather than to address major scientific concerns.

Recommendation: Minor Revision.

**********

**Summary and General Comments**

Use this section to provide overall comments, discuss strengths/weaknesses of the study, novelty, significance, general execution and scholarship. You may also include additional comments for the author, including concerns about dual publication, research ethics, or publication ethics. If requesting major revision, please articulate the new experiments that are needed.

Reviewer #1: This manuscript addresses an important and clearly under-studied topic: the sero-epidemiology of human leptospirosis in subtropical Bhutan. The topic is relevant to PLOS Neglected Tropical Diseases because leptospirosis is indeed neglected, diagnostically challenging, and highly sensitive to ecological and occupational conditions. The study also has practical public health value, since it attempts to quantify seroprevalence, describe circulating serovars, and identify behavioral and environmental risk factors in a setting where diagnostic capacity is limited. The sample size is respectable, the fieldwork appears substantial, and the effort to combine serology, spatial mapping, and risk-factor analysis is commendable. The headline finding, that roughly one in five participants had evidence of exposure by MAT, is potentially important for Bhutanese surveillance and clinical awareness.

However, despite the manuscript’s relevance and the value of the dataset, I do not think the paper is yet suitable for acceptance in its current form. My overall recommendation is rejection in the present version, with encouragement to resubmit only after major methodological, analytical, and editorial reconstruction. The main problem is not that the study question is weak; rather, the current manuscript contains too many internal inconsistencies, imprecise interpretations, and presentation problems to support publication without substantial redevelopment.

The most serious concern is methodological clarity. The authors describe MAT as the reference test and use a cutoff titre of ≥1:80, justifying this on the basis that Bhutan is “not endemic” and that surveillance sensitivity should be enhanced. That rationale is not fully convincing, especially when the manuscript simultaneously argues that leptospirosis is an underrecognized but meaningful public health problem in the same setting. A lower threshold may increase apparent seroprevalence through reduced specificity, and the paper does not adequately discuss how this decision may inflate estimates or complicate comparison with other studies. Relatedly, the manuscript interprets MAT seropositivity as evidence that “one in five people were infected,” but in a cross-sectional serosurvey this wording is too strong. Seropositivity reflects serological evidence of exposure, not necessarily current or even recent infection. The paper repeatedly moves from “exposure” to “infection” without sufficient caution, and that overstatement affects both the author summary and the discussion.

The regression analysis also raises concerns. The manuscript states that variables with p < 0.200 in univariable analysis were entered into the multivariable model, but elsewhere the wording around the screening threshold is inconsistent, and the reporting of the final model is not fully coherent. In the narrative text, the authors say that primary education was “strongly associated” with seropositivity, yet the adjusted odds ratio given is 1.42 with p = 0.318, which is clearly not statistically significant. At the same time, tabled results show other retained variables such as rural residence, secondary education, and fever in the last 12 months, some of which are not emphasized consistently in the text. This gives the impression that the interpretation of the multivariable model was not handled carefully. In addition, the manuscript does not adequately discuss model diagnostics, collinearity, missingness handling, clustering by household or community, or the implications of substituting non-responders with nearest neighboring households. That substitution procedure may introduce selection bias, particularly in a community-based serosurvey.

There are also issues with sampling and representativeness. The study uses six subtropical communities in one district and relies partly on the 2017 census for one municipality because a current household registry was unavailable. That is understandable operationally, but it weakens the claim of precise sampling representativeness. The authors should be much more restrained in describing the sample as representative, even for the district, unless they can show how the sampling frame mismatch affected inclusion probabilities. Moreover, restricting participation to Bhutanese nationals because citizenship card numbers were used as identifiers may exclude potentially high-risk populations such as migrant or mobile workers. That is a meaningful epidemiologic limitation and should be acknowledged more explicitly because it could bias seroprevalence downward or alter the apparent occupational risk structure.

Another major weakness is the interpretation of serovars. The predominance of Australis and Lailike is interesting, but the authors themselves acknowledge that the antigen panel did not include Bratislava, which was highlighted in previous Bhutan data. That omission matters. With MAT, the apparent predominant serovar can be heavily shaped by the panel selected, and without a locally validated panel or molecular confirmation the serovar discussion remains suggestive rather than definitive. The statement that no cross-reactions occurred is also somewhat surprising in MAT-based work and deserves either additional explanation or a more cautious presentation. The discussion at several points moves beyond what the data can actually establish, especially when implying transmission pathways or reservoir ecology without concurrent animal or environmental sampling.

The manuscript also suffers from substantial writing and editing problems that go beyond routine copyediting. There are repeated grammatical errors, tense inconsistencies, article misuse, awkward phrasing, and occasional factual or logical slips. For example, the introduction contains a malformed sentence around the first documented evidence of human leptospirosis in Bhutan, and elsewhere the manuscript alternates between “infection,” “exposure,” and “seropositivity” as though they were interchangeable. There are also typographical and formatting issues in the tables, such as the erroneous “16 93.3)” entry for fishery farming, inconsistent spacing around percentages and p values, and several sentences that are syntactically incomplete or imprecise. These problems materially affect readability and scientific credibility. For a journal at this level, the manuscript would need a thorough language revision, not just minor polishing.

I am also concerned by the data availability statement. The manuscript says that the original dataset is available from the corresponding author upon reasonable request, but then adds that national policy does not allow publication of the dataset on any platform. That may conflict with the journal’s data availability requirements, depending on whether a formal ethical or legal restriction can be documented and whether a controlled-access mechanism exists. As written, the statement appears weak for a PLOS journal and may become a publication barrier unless the authors provide a much more precise and policy-compliant explanation.

That said, the study has genuine strengths and should not be dismissed as unimportant. The field data are valuable, the seroprevalence estimate is potentially consequential for Bhutan, and the identification of paddy farming, unboiled water consumption, and stagnant water contact as risk correlates is biologically plausible and regionally coherent. The paper could become publishable after extensive revision if the authors reframe their claims more conservatively, clean up the analysis, clarify the sampling design, improve the description and justification of MAT thresholds, correct inconsistencies between tables and text, and substantially revise the English. But that level of work is substantial enough that I would not classify this as a paper requiring only minor or moderate revision.

My final decision is therefore to recommend rejection in the current form. This recommendation is based not on lack of relevance, but on the accumulation of methodological ambiguity, interpretive overreach, analytical inconsistency, and inadequate presentation. The underlying study appears potentially useful, but the manuscript as submitted does not yet meet the standard for acceptance

Reviewer #2: (No Response)

Reviewer #3: This manuscript presents a timely and important investigation of the sero-epidemiology of human leptospirosis in subtropical Bhutan, a setting where epidemiological data on the disease remain limited. The study addresses a significant knowledge gap by estimating human seroprevalence, identifying circulating serovars, mapping seropositive cases, and evaluating environmental and behavioral risk factors associated with exposure. Given the recent leptospirosis outbreak reported in Bhutan and the increasing influence of climate-related factors on zoonotic disease transmission, the study is highly relevant to both national and regional public health priorities.

The manuscript's major strengths include its relatively large community-based sample size, probability-based participant selection, use of the Microscopic Agglutination Test (MAT) as the reference serological assay, and comprehensive assessment of multiple environmental, occupational, and behavioral exposures. The inclusion of geospatial mapping further enhances the epidemiological value of the study. The findings provide important baseline data for future surveillance efforts and contribute to the growing evidence base on leptospirosis epidemiology in South Asia.

The study design is generally appropriate for the stated objectives, and the statistical analyses are suitable for identifying factors associated with seropositivity. The conclusions are largely supported by the results and appropriately acknowledge the limitations inherent to a cross-sectional design. The discussion effectively places the findings within the regional and global literature and highlights practical public health implications, including strengthening diagnostic capacity, surveillance systems, and targeted prevention strategies.

Several minor issues should be addressed to improve clarity and scientific rigor. The Introduction would benefit from an explicit statement of the study hypothesis. Minor inconsistencies were noted regarding the criteria used for variable selection in multivariable analysis and should be corrected. Additional clarification of model-building procedures and regression diagnostics would strengthen the analytical transparency. Furthermore, some statements in the Results and Discussion sections should be revised to ensure consistency with the reported statistical findings. Several grammatical and typographical errors were also identified and can be addressed during revision.

The limitations are appropriately discussed, including the restricted serovar panel, absence of molecular diagnostics, exclusion of animal reservoirs, and limited generalizability beyond the study area. These limitations do not substantially diminish the value of the findings but should be considered when interpreting the results.

I did not identify concerns regarding research ethics, participant consent, or publication ethics based on the information provided. Ethical approvals from the relevant institutions were obtained, and informed consent procedures were adequately described. The study appears to represent original work and does not raise concerns regarding duplicate publication.

Overall, this manuscript makes a meaningful contribution to the understanding of leptospirosis in Bhutan and provides evidence that can inform future surveillance, One Health initiatives, and public health interventions. The study is scientifically sound, addresses an important public health problem, and presents novel data from an understudied setting. The revisions required are primarily editorial and clarificatory in nature.

Recommendation: Minor Revision.

**********

PLOS authors have the option to publish the peer review history of their article (what does this mean?). If published, this will include your full peer review and any attached files.

If you choose “no”, your identity will remain anonymous but your review may still be made public.

**Do you want your identity to be public for this peer review?**  For information about this choice, including consent withdrawal, please see our Privacy Policy.

Reviewer #1: No

Reviewer #2: No

Reviewer #3: **Yes:**  Khristine Sandoval

[NOTE: If reviewer comments were submitted as an attachment file, they will be attached to this email and accessible via the submission site. Please log into your account, locate the manuscript record, and check for the action link "View Attachments". If this link does not appear, there are no attachment files.]

**Figure resubmission:**

**Reproducibility:**

To enhance the reproducibility of your results, we recommend that authors of applicable studies deposit laboratory protocols in protocols.io, where a protocol can be assigned its own identifier (DOI) such that it can be cited independently in the future. Additionally, PLOS ONE offers an option to publish peer-reviewed clinical study protocols. Read more information on sharing protocols at https://plos.org/protocols?utm_medium=editorial-email&utm_source=authorletters&utm_campaign=protocols

Attachment Submitted filename: Review comments PLOS00604.docx

# Author comment: Author response to Decision Letter 1

**DOI:** 10.1371/journal.pntd.0014821.r002

14 Aug 2026

Attachment Submitted filename: Response Letter Lepto_V3.docx

# Peer review: Decision Letter 1

**DOI:** 10.1371/journal.pntd.0014821.r003

**Authors:** Ana LTO Nascimento, Martin Wainaina

22 Sep 2026

Response to Reviewers Revised Manuscript with Track Changes Manuscript

Shaden Kamhawi

co-Editor-in-Chief

PLOS Neglected Tropical Diseases

orcid.org/0000-0003-4304-636XX

Paul Brindley

co-Editor-in-Chief

PLOS Neglected Tropical Diseases

orcid.org/0000-0003-1765-0002

**Additional Editor Comments (if provided):**

Line 110: Authors should state recommended/recognised antibiotic therapies. There is not much evidence for leptospirosis treatment using fluoroquinolones.

Line 225: The exact kit name for the ELISA kit from Gold Standard Diagnostics should be used here.

Lines 240, 244, 347 and references: Leptospira should be italicised as should all botanical names.

Line 257: Please add references as to why a cut-off of 1:80 was used.

Table 1 and in other places in the manuscript: Please change Uterecht to Utrecht, Grippotyphosa is in species L. kirschneri, Hardjoprajtno to Hardjoprajitno and Lailike to Lai-like

Statistical analysis: How was MAT serostatus defined for the logistic regressions...How was a seropositive (perhaps positive in at least one) and seronegative result (perhaps negative for all) determined considering several serovars were tested.

Lines 346 onwards: The comparisons made with other seroprevalence estimates should mention the diagnostic tests used and cut-offs.

Table 2: It seems to me “Fever in past 12 months”, “Participated in paddy plantation” and “Drink unboiled water”, "current symptoms", etc., are categorical variables, in which case presenting them as Yes/No with the numbers is better for the seroprevalence distribution tables. For something like “Fever in last 12 months” a Yes/No or Present/Absent is even necessary to interpret what 301 (61.2%) refers to.

Table 4: Same suggestion here. It would also help the reader if the significant categories were releveled/rebased to give positive (adjusted) odds ratios instead of these protective odds ratios for ease of interpretation. Also, please change “Referent” to “Reference” or “Ref” with a footnote all through the manuscript and supplementary information as it is standard.

Line 468: Oral transmission of leptospirosis is likely ineffective due to protection from saliva, intact oral mucosa and gastric acid according to evidence from experimental studies on animal models. The discussion should therefore reflect this by showing possibility of past exposure through broken skin and interrupted mucous membranes and its relationship to fetching or drinking untreated water.

Figure 3: please add confidence intervals to these estimates in the figure

**Reviewers' comments:**

**Key Review Criteria Required for Acceptance?**

As you describe the new analyses required for acceptance, please consider the following:

**Methods**

-Are the objectives of the study clearly articulated with a clear testable hypothesis stated?

-Is the study design appropriate to address the stated objectives?

-Is the population clearly described and appropriate for the hypothesis being tested?

-Is the sample size sufficient to ensure adequate power to address the hypothesis being tested?

-Were correct statistical analysis used to support conclusions?

-Are there concerns about ethical or regulatory requirements being met?

Reviewer #1: (No Response)

Reviewer #2: (No Response)

Reviewer #3: 1. Overview and Context

As the reviewer evaluating the Methods section of this manuscript on Leptospira seroepidemiology in subtropical communities (specifically in Sarpang), the overall assessment indicates that the manuscript is largely acceptable after revision. The authors have thoughtfully addressed most of the initial methodological concerns, particularly regarding the distinction between past serological evidence of exposure versus acute infection, framing appropriate inclusion/exclusion criteria, and securing the necessary ethical approvals.

However, before final acceptance and publication, attention must be paid to a few remaining technical refinements, primarily centering around statistical handling of clustered sampling and the interpretation of sparse exposure data.

2. Detailed Breakdown by Criterion

Objectives & Testable Hypothesis:

Current Status: Yes. The objectives are clear (estimating seroprevalence, identifying circulating serovars, mapping cases, assessing risk factors). A testable hypothesis linking environmental/occupational contact to MAT seropositivity has been incorporated.

Recommendation: Maintain this explicit focus in the Introduction. Ensure that the primary hypothesis remains tightly coupled with the final multivariable regression modeling.

Study Design:

Current Status: Yes. The cross-sectional seroepidemiological design is appropriate for estimating prevalence and exploring associations, with correct explicit acknowledgment that temporality cannot be established.

Recommendation: Continue to rigorously distinguish between seroprevalence/exposure and acute infection throughout the text.

Study Population & Representativeness:

Current Status: Yes, with limitations. Adults ≥18 years residing in the study areas for ≥1 year were sampled using random household selection (one adult per household across six communities).

Recommendation: Explicitly keep the noted limitation that excluding non-Bhutanese residents may impact generalizability, as migrant or foreign workers could possess distinctly different occupational exposure profiles.

Sample Size Justification:

Current Status: Yes for primary objectives; caution for subgroups. Calculations accounted for an expected prevalence of 17.6%, 5% precision, 95% confidence, a design effect of 2.0, and 5% inflation (target n=469; final analyzed n=492 with 95 MAT-positive outcomes).

Recommendation: Explicitly note that while 492 participants with 95 positive outcomes offer a reliable basis for the primary prevalence and overall model, individual subgroup categories with very few events will inevitably generate unstable estimates.

Statistical Analyses:

Current Status: Generally sound, but requires strengthening. The use of conventional logistic regression, AIC-based model comparison, forced adjustment for core variables (sex, settlement, education), VIF/GVIF diagnostics, Hosmer–Lemeshow, ROC/AUC, and Cook’s distance sensitivity analyses are thorough.

Recommendation: Address the clustered sampling structure. Because sampling occurred across six communities with household substitution, ordinary logistic regression risks underestimating standard errors due to intra-cluster correlation.

Analytical Consideration: Since there are only six communities, a random-effects mixed model may face convergence or cluster-count limitations. A cluster-robust standard error approach (at the community or sub-district level) is strongly recommended, alongside transparent descriptions of missing-data handling.

Ethical and Regulatory Compliance:

Current Status: Yes. Approved by the Research Ethics Board of Health, Bhutan, and the Institutional Review Board of the International University of Health and Welfare, alongside institutional clearances and documented written/thumb-impression consent.

Recommendation: Ensure final manuscript versions retain all clearance numbers and precise consent descriptions.

3. Key Methodological Priorities Before Acceptance

Account for Clustered Sampling:

Explicitly justify how the household/community sampling design was factored into the analysis. Implement cluster-robust standard errors if standard mixed-effects models are statistically infeasible due to the limited number of clusters (six communities).

Cautious Handling of Sparse Data & Influential Observations:

With 95 MAT-positive events against several predictors, certain categories showed extreme sensitivity (e.g., influential data point removal shifting paddy-farming odds ratios dramatically and making fever hospitalization inestimable). Do not add unnecessary extra predictors. Keep the final model parsimonious and emphasize cautious interpretation of sparse cells.

Exclusion of Diagnostic Validation (ELISA vs. MAT):

Support the decision to exclude a comprehensive ELISA-vs-MAT diagnostic accuracy analysis from this manuscript, preserving the paper's focus strictly on seroepidemiology rather than assay validation.

**********

**Results**

-Does the analysis presented match the analysis plan?

-Are the results clearly and completely presented?

-Are the figures (Tables, Images) of sufficient quality for clarity?

Reviewer #1: (No Response)

Reviewer #2: (No Response)

Reviewer #3: Does the analysis presented match the analysis plan?

Yes. The results faithfully execute the analytical framework outlined in the methods. They progress logically from descriptive statistics and seroprevalence estimates to univariable screening, AIC-based multivariable model comparison, and rigorous diagnostic checks. The decision to focus strictly on MAT-confirmed seroprevalence (while systematically presenting ELISA comparisons) and defer diagnostic validation to a separate paper is consistently maintained.

Are the results clearly and completely presented?

Yes. Participant flow is fully transparent (503 enrolled, 11 excluded due to hemolysis/insufficient volume, leaving 492 analyzed). The data clearly show a MAT-confirmed seroprevalence of 19.3%, with Australis (49%) and Lailike (13%) as predominant serovars, and geographic clustering in Gelephu Thromde. Key independent risk predictors—paddy farming (aOR: 4.74) and drinking unboiled water (aOR: 2.34)—are clearly stated.

Are the figures and tables of sufficient quality for clarity?

Yes. The workflow diagram (Fig 2) cleanly traces sample processing and denominator accountability, and the geospatial map (Fig 1) effectively illustrates community distribution with proper copyright and licensing attributions. Tabular data are structured cleanly with exact confidence intervals and p-values.

The Results section is rigorous, fully aligned with the study objectives and analysis plan, and presented with high clarity. No major modifications are required.

**********

**Conclusions**

-Are the conclusions supported by the data presented?

-Are the limitations of analysis clearly described?

-Do the authors discuss how these data can be helpful to advance our understanding of the topic under study?

-Is public health relevance addressed?

Reviewer #1: (No Response)

Reviewer #2: (No Response)

Reviewer #3: 1. Are the conclusions supported by the data presented?

Assessment: Yes.

Details:

The primary conclusion—that approximately one in five adults (19.3%) in subtropical Bhutan has serological evidence of prior Leptospira exposure—is directly derived from the MAT-confirmed prevalence data (n=95 out of 492).

The identification of key risk drivers (paddy farming and drinking unboiled water) directly matches the significant associations found in the multivariable logistic regression models.

The assertion that human exposure is more common than previously recognized is well-supported given the historical backdrop of underdiagnosis and limited surveillance data in the region.

2. Are the limitations of analysis clearly described?

Assessment: Yes.

Details:

The authors appropriately highlight the cross-sectional design, explicitly acknowledging that temporality cannot be established between the identified risk factors and the seropositive outcomes.

They transparently account for potential recall bias regarding self-reported exposures over the preceding 12 months.

Although implicit in the study setup, the text could benefit from a brief final reinforcement of the sparse category constraints and community clustering limitations evaluated in the methods, but overall, the core epidemiological limitations are honestly stated.

3. Do the authors discuss how these data can be helpful to advance our understanding of the topic under study?

Assessment: Yes.

Details:

The manuscript successfully bridges local empirical data with broader epidemiological understanding. By establishing baseline seroprevalence and mapping circulating serovars (predominantly Australis and Lailike with widespread distribution of Patoc), the study moves Bhutan past the bottleneck of sporadic outbreak-only reporting.

It provides a foundational epidemiological profile for a high-risk subtropical environment, expanding our comprehension of zoonotic spillover risks in regions where leptospirosis clinically overlaps with other endemic tropical infections like dengue, malaria, and scrub typhus.

4. Is public health relevance addressed?

Assessment: Yes, exceptionally well.

Details:

The conclusion translates the data into four concrete, actionable public health recommendations:

Educating communities on preventive behaviors.

Promoting the use of protective gear during high-risk agricultural activities (such as paddy farming).

Expanding research and surveillance capacity.

Strengthening hospital-based diagnostics to enable early antibiotic treatment and reduce severe complications.

These points directly target the structural gaps identified in the introduction and demonstrate high translational value for public health policy in Bhutan.

Reviewer Conclusion on the Conclusions

The Conclusions section is robust, tightly anchored to the empirical findings, honest about study limitations, and highly impactful regarding public health translation. No major revisions are required for this section.

**********

**Editorial and Data Presentation Modifications?**

Use this section for editorial suggestions as well as relatively minor modifications of existing data that would enhance clarity. If the only modifications needed are minor and/or editorial, you may wish to recommend “Minor Revision” or “Accept”.

Reviewer #1: (No Response)

Reviewer #2: (No Response)

Reviewer #3: Clarify Sample Size Denominator Terminology in Text:

Ensure that the distinction between the total enrolled cohort (n=503), the excluded samples due to hemolysis/inadequate volume (n=11), and the final analyzed cohort (n=492) is consistently stated across the Abstract, Methods, and Results to prevent any reader confusion regarding denominator shifts.

Refine Subgroup Data Description:

In the risk factor and multivariable results text, briefly reinforce that while the primary model is robust, individual exposure categories with sparse positive counts (e.g., specific water or high-risk sub-exposures) should be interpreted with caution. This aligns with the sensitivity analyses performed regarding influential data points.

Geospatial and Labeling Polish (Figure 1):

Ensure that the boundaries and text annotations on the map (Figure 1) remain fully legible when formatted into the final multi-column journal layout, verifying that district names and legend items contrast cleanly against the Esri World Imagery basemap.

Minor Copyediting & Grammar Tweaks:

Introduction/Methods: Check minor phrasing items, such as ensuring uniform verb tense when describing the execution of the 2024 field survey versus subsequent laboratory processing.

Conclusion/Public Health Recommendations: The four bulleted public health interventions are strong and actionable; ensure they mirror the precise wording used between the abstract summary and the final concluding paragraphs for maximum rhetorical impact.

Overall Recommendation: The manuscript is in exceptional shape. Implementing these minor editorial polishes will ensure complete clarity for the readership of PLOS Neglected Tropical Diseases.

**********

**Summary and General Comments**

Use this section to provide overall comments, discuss strengths/weaknesses of the study, novelty, significance, general execution and scholarship. You may also include additional comments for the author, including concerns about dual publication, research ethics, or publication ethics. If requesting major revision, please articulate the new experiments that are needed.

Reviewer #1: (No Response)

Reviewer #2: (No Response)

Reviewer #3: 1. Overview and Evaluation Verdict

This manuscript presents a rigorous, timely, and epidemiologically sound investigation into the seroprevalence and associated risk factors of human leptospirosis in subtropical Bhutan. Utilizing a probability-proportional-to-size (PPS) sampling framework across six communities in the Sarpang district, the authors successfully bridge a critical knowledge gap in a region where leptospirosis is heavily under-recognized due to diagnostic constraints and clinical overlap with other endemic tropical infections (such as dengue, malaria, and scrub typhus).

Overall, the manuscript exhibits high scholastic quality, transparent reporting, and exceptional public health relevance. The study is well-executed, the laboratory diagnostics (combining IgM/IgG ELISA screening with the gold-standard Microscopic Agglutination Test across a robust 13-serovar panel) are meticulously performed, and the statistical framework is sophisticated—incorporating multiple multivariable specifications, AIC model comparisons, collinearity diagnostics, and sensitivity analyses.

2. Novelty and Significance

Filling a Critical Evidence Gap: To the best of our knowledge, this is the first comprehensive human seroprevalence study conducted in this high-risk subtropical zone of Bhutan. Historically, national surveillance has relied on passive reporting or outbreak-driven tracking (such as the significant 2025 outbreak). Establishing a baseline seroprevalence of 19.3% (approximately one in five adults) fundamentally reshapes our understanding of disease endemicity, demonstrating that asymptomatic or mild, unrecognized exposure is far more common than previously appreciated.

Actionable Epidemiological Mapping: The identification of predominant serovars (Australis at 49% and Lailike at 13%) alongside geospatial clustering within Gelephu Thromde provides local health authorities with invaluable targets for vector/reservoir control and resource allocation.

Translational Public Health Value: The study avoids purely academic abstraction by directly linking its empirical findings—specifically paddy farming and drinking unboiled water as independent risk drivers—to four concrete, highly actionable public health recommendations (community education, protective occupational gear, diagnostic expansion, and regional surveillance strengthening).

3. Major Strengths of the Study

Rigorous Sampling Design: The implementation of PPS sampling based on district registries, paired with an a priori sample size calculation accounting for a design effect (2.0) and non-response inflation, ensures strong internal validity and representativeness of the target population.

Methodological Transparency: The analytical approach is exemplary. By testing multiple model selection strategies (bidirectional, backward, forced sociodemographic control, and LASSO cross-validation) and rigorously reporting diagnostic checks (VIF/GVIF, Hosmer-Lemeshow, ROC/AUC, and Cook's distance), the authors demonstrate exceptional analytical rigor.

Pragmatic Diagnostic Scope: The authors made the correct methodological decision to focus primarily on MAT-confirmed seroprevalence for risk factor modeling while systematically presenting ELISA comparisons, and properly deferring a full ELISA-vs-MAT diagnostic validation to a separate dedicated manuscript.

4. Limitations and Areas for Careful Interpretation

As transparently acknowledged by the authors:

Cross-Sectional Constraints: The cross-sectional design inherently precludes establishing temporality between self-reported exposures (over the preceding 12 months) and MAT seropositivity.

Recall Bias & Sparse Categories: Self-reported behavioral exposures are susceptible to recall bias. Furthermore, certain sub-exposures suffered from sparse positive event counts, meaning that multivariable effect sizes (such as those observed in sensitivity checks upon removing influential observations) must be interpreted with appropriate conservatism.

Clustered Sampling Considerations: While ordinary logistic regression was thoroughly evaluated, the authors should ensure that standard error estimations appropriately account for intra-cluster correlation across the six sampled communities, or clearly state this as a minor analytical boundary.

5. Ethics, Regulatory, and Publication Compliance

Research Ethics: The study demonstrates full compliance with ethical and regulatory requirements, holding formal approvals from the Research Ethics Board of Health (REBH), Bhutan, and the Institutional Review Board of the International University of Health and Welfare (IUHW), Japan, alongside administrative site clearances.

Informed Consent: Ethical acquisition of written consent (including witnessed thumb impressions for non-literate participants) is fully documented.

Data Availability: The manuscript complies with open science standards, providing clear statements regarding underlying data access through institutional contact points and upcoming national repositories.

6. Final Recommendation

The manuscript is exceptionally well-prepared and meets all necessary criteria for publication in PLOS Neglected Tropical Diseases. No major new experiments or data collection are required.

Final Verdict: Accept (or Minor Revision solely to accommodate the final minor editorial polishes regarding sample denominator clarity and cluster-robust standard error text documentation).

**********

PLOS authors have the option to publish the peer review history of their article (what does this mean?). If published, this will include your full peer review and any attached files.

If you choose “no”, your identity will remain anonymous but your review may still be made public.

**Do you want your identity to be public for this peer review?**  For information about this choice, including consent withdrawal, please see our Privacy Policy.

Reviewer #1: No

Reviewer #2: No

Reviewer #3: No

**Figure resubmission:**

**Reproducibility:** -->To enhance the reproducibility of your results, we recommend that authors of applicable studies deposit laboratory protocols in protocols.io, where a protocol can be assigned its own identifier (DOI) such that it can be cited independently in the future. Additionally, PLOS ONE offers an option to publish peer-reviewed clinical study protocols. Read more information on sharing protocols at https://plos.org/protocols?utm_medium=editorial-email&utm_source=authorletters&utm_campaign=protocols-->?>

# Author comment: Author response to Decision Letter 2

**DOI:** 10.1371/journal.pntd.0014821.r004

1 Oct 2026

Attachment Submitted filename: Response_Letter_Lepto_R2_V2.docx

# Editor report: Decision Letter 2

**DOI:** 10.1371/journal.pntd.0014821.r005

**Authors:** Ana LTO Nascimento, Martin Wainaina

6 Oct 2026

Response to Reviewers Revised Manuscript with Track Changes Manuscript

We look forward to receiving your revised manuscript.

Shaden Kamhawi

co-Editor-in-Chief

PLOS Neglected Tropical Diseases

orcid.org/0000-0003-4304-636XX

Paul Brindley

co-Editor-in-Chief

PLOS Neglected Tropical Diseases

orcid.org/0000-0003-1765-0002

**Additional Editor Comments (if provided):**

However, categorical variables have to be presented as standard with all categories they contain, and a reference category be identified where applicable.

Redundancies and being succinct on this does not apply here as the data must be presented clearly to all readers.

**Reviewers' comments:** **Figure resubmission:**

**Reproducibility:** -->To enhance the reproducibility of your results, we recommend that authors of applicable studies deposit laboratory protocols in protocols.io, where a protocol can be assigned its own identifier (DOI) such that it can be cited independently in the future. Additionally, PLOS ONE offers an option to publish peer-reviewed clinical study protocols. Read more information on sharing protocols at https://plos.org/protocols?utm_medium=editorial-email&utm_source=authorletters&utm_campaign=protocols-->?>

# Author comment: Author response to Decision Letter 3

**DOI:** 10.1371/journal.pntd.0014821.r006

8 Oct 2026

Attachment Submitted filename: Response to Reviewers.docx

# Editor report: Decision Letter 3

**DOI:** 10.1371/journal.pntd.0014821.r007

**Authors:** Ana LTO Nascimento, Martin Wainaina

15 Oct 2026

We are pleased to inform you that your manuscript 'Seroepidemiology of human leptospirosis in subtropical Bhutan, 2024' has been provisionally accepted for publication in PLOS Neglected Tropical Diseases.

Before your manuscript can be formally accepted you will need to complete some formatting changes, which you will receive in a follow up email. A member of our team will be in touch with a set of requests.

Please note that your manuscript will not be scheduled for publication until you have made the required changes, so a swift response is appreciated.

IMPORTANT: The editorial review process is now complete. PLOS will only permit corrections to spelling, formatting or significant scientific errors from this point onwards. Requests for major changes, or any which affect the scientific understanding of your work, will cause delays to the publication date of your manuscript.

Should you, your institution's press office or the journal office choose to press release your paper, you will automatically be opted out of early publication. We ask that you notify us now if you or your institution is planning to press release the article. All press must be co-ordinated with PLOS.

Thank you again for supporting Open Access publishing; we are looking forward to publishing your work in PLOS Neglected Tropical Diseases.

Best regards,

Martin Wainaina

Academic Editor

PLOS Neglected Tropical Diseases

Ana LTO Nascimento

Section Editor

PLOS Neglected Tropical Diseases

Shaden Kamhawi

co-Editor-in-Chief

PLOS Neglected Tropical Diseases

orcid.org/0000-0003-4304-636XX

Paul Brindley

co-Editor-in-Chief

PLOS Neglected Tropical Diseases

orcid.org/0000-0003-1765-0002

***********************************************************

# Editor report: Acceptance letter

**DOI:** 10.1371/journal.pntd.0014821.r008

**Authors:** Ana LTO Nascimento, Martin Wainaina

Dear Mr Tamang,

We are delighted to inform you that your manuscript, "Seroepidemiology of human leptospirosis in subtropical Bhutan, 2024," has been formally accepted for publication in PLOS Neglected Tropical Diseases.

We have now passed your article onto the PLOS Production Department who will complete the rest of the publication process. All authors will receive a confirmation email upon publication.

The corresponding author will soon be receiving a typeset proof for review, to ensure errors have not been introduced during production. Please review the PDF proof of your manuscript carefully, as this is the last chance to correct any scientific or type-setting errors. Please note that major changes, or those which affect the scientific understanding of the work, will likely cause delays to the publication date of your manuscript. Note: Proofs for Front Matter articles (Editorial, Viewpoint, Symposium, Review, etc...) are generated on a different schedule and may not be made available as quickly.

Soon after your final files are uploaded, the early version of your manuscript will be published online unless you opted out of this process. The date of the early version will be your article's publication date. The final article will be published to the same URL, and all versions of the paper will be accessible to readers.

For Research Articles, you will receive an invoice from PLOS for your publication fee after your manuscript has reached the completed accept phase. If you receive an email requesting payment before acceptance or for any other service, this may be a phishing scheme. Learn how to identify phishing emails and protect your accounts at https://explore.plos.org/phishing.

Thank you again for supporting open-access publishing; we are looking forward to publishing your work in PLOS Neglected Tropical Diseases.

Best regards,

Shaden Kamhawi

co-Editor-in-Chief

PLOS Neglected Tropical Diseases

Paul Brindley

co-Editor-in-Chief

PLOS Neglected Tropical Diseases
